# Reconstructing relative transmission rates in Bayesian phylodynamics: Two-fold transmission advantage of Omicron in Berlin, Germany during December 2021

**DOI:** 10.1093/ve/vead070

**Published:** 2023-11-29

**Authors:** Ariane Weber, Sanni Översti, Denise Kühnert

**Affiliations:** Transmission, Infection, Diversification & Evolution Group (tide), Max Planck Institute of Geoanthropology, Kahlaische Strasse 10, Jena, Thuringia 07745, Germany; Max Planck Institute for Evolutionary Anthropology, Deutscher Platz 6, Leipzig, Saxony 04103, Germany; Transmission, Infection, Diversification & Evolution Group (tide), Max Planck Institute of Geoanthropology, Kahlaische Strasse 10, Jena, Thuringia 07745, Germany; Max Planck Institute for Evolutionary Anthropology, Deutscher Platz 6, Leipzig, Saxony 04103, Germany; Centre for Artificial Intelligence in Public Health Research, Robert Koch Institute, Ludwig-Witthöft-Straße 14, Wildau, Brandenburg 15745, Germany

**Keywords:** Bayesian phylodynamics, birth–death, simulations, SARS-CoV-2, transmission advantage, reproductive number

## Abstract

Phylodynamic methods have lately played a key role in understanding the spread of infectious diseases. During the coronavirus disease (COVID-19) pandemic, large scale genomic surveillance has further increased the potential of dynamic inference from viral genomes. With the continual emergence of novel severe acute respiratory syndrome coronavirus type 2 (SARS-CoV-2) variants, explicitly allowing transmission rate differences between simultaneously circulating variants in phylodynamic inference is crucial. In this study, we present and empirically validate an extension to the BEAST2 package birth–death skyline model (BDSKY), BDSKY$\lambda $, which introduces a scaling factor for the transmission rate between independent, jointly inferred trees. In an extensive simulation study, we show that BDSKY$\lambda $ robustly infers the relative transmission rates under different epidemic scenarios. Using publicly available genome data of SARS-CoV-2, we apply BDSKY$\lambda $ to quantify the transmission advantage of the Omicron over the Delta variant in Berlin, Germany. We find the overall transmission rate of Omicron to be scaled by a factor of two with pronounced variation between the individual clusters of each variant. These results quantify the transmission advantage of Omicron over the previously circulating Delta variant, in a crucial period of pre-established non-pharmaceutical interventions. By inferring variant- as well as cluster-specific transmission rate scaling factors, we show the differences in transmission dynamics for each variant. This highlights the importance of incorporating lineage-specific transmission differences in phylodynamic inference.

## Introduction

Recent epidemic outbreaks, such as those caused by HIV, Ebolavirus, and severe acute respiratory syndrome coronavirus type 2 (SARS-CoV-2), have proven the relevance of phylodynamic models for inferring viral transmission dynamics and predicting disease spread (Dennis et al. 2014; Holmes et al. 2016; Attwood et al. 2022). In a phylodynamic framework, properties of key epidemiological parameters, such as the growth rate and reproductive number, can be deducted directly from phylogenies reconstructed based on viral genomic data (Grenfell et al. 2004; Pybus and Rambaut 2009; Kühnert, Wu, and Drummond 2011; Attwood et al. 2022). Evolutionary relationships of pathogen sequences are commonly inferred in combination with a coalescent or birth–death model as the underlying tree generating process. The coalescent is a probabilistic model that traces the ancestry of a study population backwards in time while the birth–death process is a stochastic model that reconstructs the evolutionary process forward in time. For details on the model differences see for example Boskova, Bonhoeffer, and Stadler 2014; Volz and Frost 2014.

To infer phylogenetic trees, the Bayesian statistical framework has become extensively used as it enables the incorporation of complex demographic models while simultaneously accounting for phylogenetic uncertainty. Currently, one of the most preeminent software platforms to conduct Bayesian phylodynamic analysis is Bayesian Evolutionary Analysis by Sampling Trees (BEAST2), which employs a Markov Chain Monte Carlo (MCMC) algorithm to draw samples from the posterior distributions of the parameters under scrutiny (Bouckaert et al. 2019). Within the BEAST2 framework, numerous population dynamic models have been developed (Pybus, Rambaut, and Harvey 2000; Drummond et al. 2005; Stadler et al. 2013). In the birth–death skyline model (BDSKY) (Stadler et al. 2013), changes in population size over time are described through birth (transmission), death (recovery), and sampling (sequencing) events. Through time, these events occur randomly, governed by their respective rate parameters: the transmission rate $\lambda $, recovery rate $\mu $, and sampling rate $\psi $. These rates are piecewise constant, allowing for changes in the transmission dynamics through time.

Since its emergence in late 2019, SARS-CoV-2 has been causing fast-growing outbreaks globally. Over the course of the pandemic, different variants have shown to bear highly variable transmission dynamics. SARS-CoV-2 lineages that show increased transmissibility, disease severity, or decreased response to interventions at a degree of public health significance have been designated as variants of concern (VOCs) by the World Health Organization. During the first three years of the pandemic new VOCs have emerged repeatedly, displacing each other. It is evident that the accumulation of advantageous mutations and subsequent positive selection (Martin et al. 2021) has substantially impacted the properties of SARS-CoV-2. As a result of these genomic alterations, some strains became more effectively transmitted than others. One example for this is the Omicron variant, which was first detected in South Africa in November 2021 and had at the end of December 2021 virtually replaced all preceding lineages in most parts of the world (Viana et al. 2022). The associated parent Pango lineage (Rambaut et al. 2020) B.1.1.529 was identified to carry more adaptive mutations than any of the previous VOCs, leading to exceptionally high levels of transmission (Martin et al. 2022).

As in the case of SARS-CoV-2, individual lineages of a pathogen may demonstrate highly distinctive transmission dynamics. This variation needs to be accounted for when epidemiological parameters are reconstructed. In this paper, we introduce the BDSKYλ model, an extension to the existing birth–death skyline model that allows the estimation of relative transmission rates between jointly inferred trees. This enables the inference of transmission differences between outbreak clusters relative to one baseline rate. Through a simulation study, we empirically validate and evaluate the performance of this approach. We apply the BDSKYλ model to SARS-CoV-2 data sampled in the federal state Berlin, Germany, including the capital city Berlin, during December 2021 to evaluate the local transmission difference between the Delta and Omicron variant. Through the estimation of variant-specific properties, our results quantify the substantially higher transmissibility of the Omicron over the Delta variant, even in the special case of pre-established strict non-pharmaceutical interventions (NPIs). As, however, extrinsic factors can highly influence the observed transmission dynamics, we explore the variability of these between same-variant clusters to evaluate the demographic impact. Despite the overall substantially higher transmissibility, we find pronounced variability in the cluster-specific transmission rates, overlapping between variants.

## Methods

### Birth–death skyline model with transmission rate ratios

The BDSKYλ model is an extension to the previously published birth–death skyline model and its Bayesian inference implementation in the BEAST2 package BDSKY. It describes the joint analysis of multiple trees which share a baseline transmission rate that is scaled individually for each tree. Assuming independence between trees, the full posterior is calculated using the product of all tree-specific phylogenetic likelihoods and tree generating probabilities. In the latter, the transmission rate is replaced by the product of the baseline transmission rate ${\lambda _{base}}$ and its tree-specific scaling factor ${r_\lambda }$. When considering the alternative model formulation, in terms of epidemiological parameters, the baseline effective reproductive number ${R_{base}}$ will depend on the product of these two. Supplementary text S1 contains a list of definitions for terms used in this study, and Supplementary text S2 a more detailed description of the BDSKYλ model. We follow the mathematical notation introduced for the birth–death skyline model (Stadler et al. 2013). The more technical terms used throughout this work, we define as follows: a *transmission cluster* is a group of infected individuals from a panmictic, spatially defined population, with homogenous transmission dynamics starting from the index patient of the cluster. The *transmission tree* is a binary tree representation of a transmission process, here assumed to arise from a birth–death sampling process. The phylogenetic tree is reconstructed from viral genetic sequences. A genetic cluster presents a set of viral sequences that is selected based on genetic differences. The latter we use as an approximation of transmission clusters. The method is available on GitHub in the BEAST2 package BDSKY. As it only introduces one additional new parameter for each tree class, the run time of BDSKYλ is not substantially different to a BDSKY analysis.

### Simulation study

To validate BDSKYλ, we simulated transmission trees under a birth–death-sampling model and sequence data along these trees. Datasets were then analysed using the BDSKYλ model in BEAST v.2.6.6 to confirm that parameters included in the model can be accurately re-estimated. Trees were simulated under 10 different scenarios with the MASTER package (Vaughan and Drummond 2013). For each scenario, we simulated 100 replicates after discarding epidemics that died out before the requested number of tips (${N_{tips}}$) was reached. Sequence alignments were simulated under the HKY model (Hasegawa, Kishino, and Yano 1985) by assuming a rate of 0.0008 substitutions/site/year (Ghafari et al. 2020) and a sequence length of 30,000 bp, corresponding to the genome size of SARS-CoV-2 (Wu et al. 2020). The simulation scenarios were chosen to resemble transmission dynamics characteristic for SARS-CoV-2, with properties extendable to many other measurably evolving pathogen populations. We accounted for higher and lower transmission potential, decreasing transmission due to interventions, temporal lags in detection, differences in cluster sizes, and variation in the number of outbreak clusters.

For all simulation scenarios, we assumed the rate to become non-infectious, $\delta = 36.5$ years^−1^ (Supplementary text S3). In simulation scenarios 1–7, 9, and 10, transmission rates were selected based on previous work (Liu et al. 2020; Liu and Rocklöv 2021). With ${R_{base}} = 3.0$, the transmission rate ratio was set to either ${r_{\lambda ,low}} = 1.0\,$or ${r_{\lambda ,high}} = 1.666$ (i.e. $R = 3.0$ and $R = 5.0$, respectively). In scenario 7, we simulated a piecewise decline in the transmission rate by setting ${R_{base,\,t1}} = 3.0$ and ${R_{base,\,t2}} = 2.3$. In scenario 8, we assumed the same but with lower transmission, i.e. ${R_{base,\,t1}} = 1.17$, ${R_{base,\,t2}} = 0.9$, ${r_{\lambda ,low}} = 1.0$, and ${r_{\lambda ,high}} = 1.6$. In scenarios 9 and 10, we additionally assessed the model’s sensitivity for and behaviour with some variation within transmission rate ratio classes. Again, ${R_{base}} = 3.0$ was assumed whereas trees representing the ${r_{\lambda ,low}}$ class were simulated by assuming $r_{\lambda,low}=0.8-1.2$ and trees belonging to the ${r_{\lambda ,high}}$ class were reconstructed by assuming $r_{\lambda,high}=1.4-1.8$. Furthermore, in scenarios 2–8, for particular transmission trees, the onset of the sampling process was delayed as a detection lag of an average 14 days has been identified (du Plessis et al. 2021). The main differences between simulation scenarios are presented in Table 1. For details, see Supplementary text S3 and Supplementary Table S1.

**Table 1.** Overview of the main differences between simulation scenarios. Each simulation was run until a specific number of tips (${N_{tips}}$) was reached.

Scenario	Transmission parameters	Number of simulated transmission trees	Number of sampled tips	Bayesian inference
1	${R_{base}} = 3.0$ ${r_{\lambda ,low\,}} = 1.0$ ${r_{\lambda ,high}} = 1.666$	5	${N_{tips}} = 25\,$ or ${N_{tips}} = 250$	${r_\lambda }$ and s inferred independently for each transmission tree
2	${R_{base}} = 3.0$ ${r_{\lambda ,low\,}} = 1.0$ ${r_{\lambda ,high}} = 1.666$	5	${N_{tips}} = 25$	${r_\lambda }$ and s inferred independently for each transmission tree
3	${R_{base}} = 3.0$ ${r_{\lambda ,low\,}} = 1.0$ ${r_{\lambda ,high}} = 1.666$	5	${N_{tips}} = 100$	${r_\lambda }$ and s inferred independently for each transmission tree
4	${R_{base}} = 3.0$ ${r_{\lambda ,low\,}} = 1.0$ ${r_{\lambda ,high}} = 1.666$	6	${N_{tips}} = 50$	${r_\lambda }$ and s inferred jointly for trees with ${r_{\lambda ,low\,}}$ and jointly for trees with ${r_{\lambda ,high}}$
5	${R_{base}} = 3.0$ ${r_{\lambda ,low\,}} = 1.0$ ${r_{\lambda ,high}} = 1.666$	6	${N_{tips}} = 10\,,$ ${N_{tips}} = 20$ or ${N_{tips}} = 50$	${r_\lambda }$ and s inferred jointly for trees with ${r_{\lambda ,low\,}}$ and jointly for trees with ${r_{\lambda ,high}}$
6	${R_{base}} = 3.0$ ${r_{\lambda ,low\,}} = 1.0$ ${r_{\lambda ,high}} = 1.666$	36	${N_{tips}} = 2\,$ or ${N_{tips}} = 50$	${r_\lambda }$ and s inferred jointly for trees with ${r_{\lambda ,low\,}}$ and jointly for trees with ${r_{\lambda ,high}}$
7	${R_{base,t1}} = 3.0$ ${R_{base,t2}} = 2.3$ ${r_{\lambda ,low\,}} = 1.0$ ${r_{\lambda ,high}} = 1.666$	6	${N_{tips}} = 50$	${r_\lambda }$ and s inferred jointly for trees with ${r_{\lambda ,low\,}}$ and jointly for trees with ${r_{\lambda ,high}}$
8	${R_{base,t1}} = 1.17$ ${R_{base,t2}} = 0.9$ ${r_{\lambda ,low\,}} = 1.0$ ${r_{\lambda ,high}} = 1.6$	6	${N_{tips}} = 10\,,$ ${N_{tips}} = 20$ or ${N_{tips}} = 50$	${r_\lambda }$ and s inferred jointly for trees with ${r_{\lambda ,low\,}}$ and jointly for trees with ${r_{\lambda ,high}}$
9	${R_{base}} = 3.0$ $r_{\lambda,low{}}=0.8-1.2$ $r_{\lambda,high}=1.4-1.8$	10	${N_{tips}} = 25$	${r_\lambda }$ and s inferred independently for each transmission tree
10	${R_{base}} = 3.0$ $r_{\lambda,low{}}=0.8-1.2$ $r_{\lambda,high}=1.4-1.8$	10	${N_{tips}} = 25$	${r_\lambda }$ and s inferred jointly for trees with ${r_{\lambda ,low\,}}$ and jointly for trees with ${r_{\lambda ,high}}$

For the Bayesian inference, a strict molecular clock model was used with a fixed rate of 0.0008 substitutions/site/year. The rate to become non-infectious was fixed to its true value ($\delta = $ 36.5 years^−1^). Depending on the scenario, transmission rate ratio (${r_\lambda }$) and sampling proportion ($s$) were either estimated independently for each transmission tree or jointly for trees belonging to the same tree class (Table 1). For all scenarios, one ${r_\lambda }$ parameter was fixed to its true value to inform ${R_{base}}$ and serve as the reference for the relative transmission rate ratios. Details are given in Supplementary text S3 and Supplementary Table S1. The MCMC chain length was originally set to 3 × 10^7^–10^8^ steps. In scenario 1, for those simulation replicates for which effective sample size (ESS) values after the initial analysis were below 200 but the chain was close to convergence, the MCMC chain was allowed to run for additional 10^8^ steps. For all the simulation scenarios, the following metrics were calculated for the inferred parameters of interest (${R_{base}}$, ${r_\lambda }$, and $s$) to evaluate the model performance: median, relative error, relative bias, relative highest posterior density interval (HPDI) width, and the 95 per cent HPD accuracy. Replicates for which not all estimated parameters yielded a minimum ESS of 200 were excluded.

### SARS-CoV-2 data analysis

The BDSKYλ implementation allows us to characterize the initial epidemic spread of the Omicron variant compared to the Delta variant in Berlin. We downloaded from the Global Initiative on Sharing All Influenza Data (GISAID) (Elbe and Buckland-Merrett 2017; Shu and McCauley 2017; Khare et al. 2021) on 29 January 2022 all sequences that were complete and collected between 30 November 2021 and 31 December 2021, excluding low coverage sequences (>5 per cent of ‘N’s), together with their metadata. This time frame starts with the collection of the first Omicron-assigned sequence in Berlin and spans the first month of subsequent spread. The initial data set comprised 1887 sequences in total (Supplementary Table S2), out of which we built a multiple sequence alignment (MSA) by aligning them to the reference genome MN908947.3 using the *keeplength* and *addfragments* method in MAFFT v7.453 (Katoh and Standley 2013). To mask potential erratic positions in the genome that might interfere with phylogenetic inference, we followed recommendations in replacing specific genomic positions with an uninformative ‘N’ (De Maio et al. 2020). Grouping by the assigned Pango lineages (Rambaut et al. 2020) of each sequence, contained in the metadata, we identified genetic clusters within each group with ClusterPicker1.2.5 (Ragonnet-Cronin et al. 2013). In ClusterPicker, genetic clusters can be defined based on a maximum genetic distance and/or tree branching support values. As ClusterPicker requires as an input both MSA and phylogenetic tree, we constructed maximum likelihood substitution trees with IQ-Tree 2.1.3 (Minh et al. 2020) individually for each Pango lineage. We then applied ClusterPicker with an initial and main support threshold of zero and a genetic distance threshold of 0$.$041 per cent using the p-distance for A, C, G, and T sites only. The genetic distance threshold was chosen as an upper bound of expected within-lineage substitutions over one month (Tay et al. 2022) (see also Supplementary text S4). Excluding clusters of less than four sequences, we set up a first BDSKYλ analysis using BEAST v2.6.6 with the remaining 1213 sequences in total. For each genetic cluster, we inferred a separate tree, linking the substitution model, baseline reproductive number, and rate to become non-infectious between all trees while linking the transmission rate ratio as well as sampling proportion only for same-variant clusters. The substitution process was described using an HKY model with a fixed rate of 0.0008 substitutions/site/year. As for the simulations, for all clusters, the rate to become non-infectious was fixed to 36.5 years^−1^ over the whole time span. The baseline reproductive number was allowed to change on 30 November 2021 and a Lognormal(0.0,16.0) prior distribution was used in both intervals. The date was chosen as it roughly marks the implementation of more strict NPIs in Berlin. The transmission rate ratio was fixed to 1.0 for all Delta-associated clusters and estimated using a Lognormal(0.0,1.0) prior distribution for Omicron-associated clusters. The sampling proportion was set to 0.0 before 30 November 2021 and estimated afterwards for both Delta- and Omicron-associated clusters with Beta(40.0,960.0) as strict prior information. The mean value of the prior distribution was set to match the extrapolated expected fraction of variant-specific sequences in the data set from the calculated fractions of variants in variant-specific PCR tests in Berlin (https://www.rki.de/DE/Content/InfAZ/N/Neuartiges_Coronavirus/Situationsberichte/Wochenbericht/Wochenberichte_Tab.html, last visited 11 July 2023). MCMC chains were run for 10^8^ steps and convergence was assessed in Tracer v1.7.1 (Rambaut et al. 2018) by ESS values over 200 for all estimated parameters. The resulting posterior distributions of trees were summarized into maximum clade credibility trees using TreeAnnotator v2.6.0. Custom R and python scripts were used to summarise the numerical parameter distributions and to visualize the results, lineage-through-time plots were calculated using functions included in the ape package (Paradis and Schliep 2019). The second analysis presented in the main text was set up in the same way, except that all clusters with less than 20 sequences were excluded. This way, the transmission rate ratio could be inferred individually for each included cluster, with the ratio for the biggest Delta-associated cluster set to one. A range of sensitivity analyses dealing with variant-specific rates to become non-infectious, differences in the sampling scheme and minimal analysed cluster size are outlined in Supplementary text S5.

## Results

### Simulation study

We tested BDSKYλ through a simulation study, the results of which are summarized in Tables 2–5 and in Supplementary Figures S1–S4. In scenarios 1–3 and 9, ${r_\lambda }$ and *s* were estimated individually for each transmission tree. For the rest of the scenarios, ${r_\lambda }$ and *s* were jointly estimated for trees sharing the same transmission dynamics. In scenarios 4–6, we further tested the impact of the number of taxa on the model performance whereas in scenario 7 the effect of a change in the transmission rate was evaluated. In scenario 8, we examined how effectively the model recovers parameters when an epidemic can be considered declining ($R \lt $ 1.0). Lastly, in scenarios 9 and 10, we explored the robustness of our model to estimate modest variation in the transmission rates of linked trees.

**Table 2.** Results from simulation scenarios 1–3. For Bayesian inference, r_λ,3_ was fixed to its true value (r_λ,3_ = 1.0) for all scenarios. For scenario 1, out of 100 simulation replicates, 84 yielded ESS > 200 for all estimated parameters. For scenario 2, all simulation replicates yielded ESS > 200 for all the parameters, whereas for scenario 3 in total 96 replicates yielded ESS > 200 for all estimated parameters.

Scenario	Parameter	Truth	Median	Relative error	Relative bias	Relative HPD width	95% HPD accuracy
1	R_base_	3.0	3.027	0.063	0.009	0.394	96
	r_λ,1_	1.0	1.013	0.103	0.013	0.561	98
	r_λ,2_	1.0	1.014	0.099	0.014	0.564	96
	r_λ,3_	1.0	1.0 (fixed)	–	–	–	–
	r_λ,4_	1.0	1.038	0.102	0.037	0.580	96
	r_λ,5_	1.666	1.639	0.069	−0.016	0.421	99
	s_1_	0.01	0.010	0.079	−0.035	1.137	100
	s_2_	0.01	0.009	0.073	−0.045	1.126	100
	s_3_	0.01	0.010	0.076	−0.030	1.137	100
	s_4_	0.01	0.010	0.072	−0.036	1.135	100
	s_5_	0.001	0.001	0.552	0.404	2.764	98
2	R_base_	3.0	3.061	0.080	0.020	0.402	97
	r_λ,1_	1.0	0.997	0.109	−0.003	0.554	95
	r_λ,2_	1.0	1.002	0.108	0.002	0.558	96
	r_λ,3_	1.0	1.0 (fixed)	–	–	–	–
	r_λ,4_	1.0	0.991	0.100	−0.009	0.550	95
	r_λ,5_	1.666	1.574	0.107	−0.055	0.506	92
	s_1_	0.01	0.010	0.072	−0.038	1.132	100
	s_2_	0.01	0.010	0.083	−0.015	1.147	100
	s_3_	0.01	0.010	0.072	−0.020	1.145	100
	s_4_	0.01	0.010	0.065	−0.010	1.153	100
	s_5,1_	0.0	0.0 (fixed)	–	–	–	–
	s_5,2_	0.01	0.010	0.051	−0.022	1.172	100
3	R_base_	3.0	3.013	0.039	0.004	0.208	96
	r_λ,1_	1.0	1.006	0.054	0.006	0.294	99
	r_λ,2_	1.0	1.001	0.053	7e-04	0.293	96
	r_λ,3_	1.0	1.0 (fixed)	–	–	–	–
	r_λ,4_	1.0	1.008	0.057	0.008	0.295	97
	r_λ,5_	1.666	1.630	0.050	−0.022	0.299	98
	s_1_	0.01	0.01	0.108	−0.027	1.012	98
	s_2_	0.01	0.01	0.115	−0.014	1.021	100
	s_3_	0.01	0.01	0.112	−0.014	1.019	100
	s_4_	0.01	0.01	0.117	−0.022	1.015	100
	s_5,1_	0.0	0.0 (fixed)	–	–	–	–
	s_5,2_	0.01	0.01	0.099	−0.030	1.093	100

**Table 3.** Results from simulation scenarios 4–6. Transmission rate ratio and sampling proportions were jointly estimated for transmission trees belonging to the same tree class. For Bayesian inference, r_λ,1_ was fixed to its true value (r_λ,1_ = 1.0). For scenarios 4 and 5, all simulation replicates yielded ESS > 200 for all estimated parameters. For scenario 6, in total 98 replicates yielded ESS > 200 for all the parameters.

Scenario	Parameter	Truth	Median	Relative error	Relative bias	Relative HPD width	95% HPD accuracy
4	R_base_	3.0	2.983	0.032	−0.006	0.174	95
	r_λ,1_	1.0	1.0 (fixed)	–	–	–	–
	r_λ,2_	1.666	1.630	0.046	−0.022	0.246	97
	s_1,1_	0.0	0.0 (fixed)	–	–	–	–
	s_1,2_	0.01	0.012	0.227	0.206	1.082	99
	s_2,1_	0.0	0.0 (fixed)	–	–	–	–
	s_2,2_	0.01	0.010	0.122	0.005	1.080	100
5	R_base_	3.0	2.983	0.051	−0.006	0.233	95
	r_λ,1_	1.0	1.0 (fixed)	–	–	–	–
	r_λ,2_	1.666	1.598	0.071	−0.041	0.311	91
	s_1,1_	0.0	0.0 (fixed)	–	–	–	–
	s_1,2_	0.01	0.013	0.263	0.259	1.212	100
	s_2,1_	0.0	0.0 (fixed)	–	–	–	–
	s_2,2_	0.01	0.010	0.082	−0.019	1.120	100
6	R_base_	3.0	2.694	0.102	−0.102	0.191	44
	r_λ,1_	1.0	1.0 (fixed)	–	–	–	–
	r_λ,2_	1.666	1.6137	0.062	−0.031	0.297	93
	s_1,1_	0.0	0.0 (fixed)	–	–	–	–
	s_1,2_	0.01	0.012	0.169	0.157	1.127	100
	s_2,1_	0.0	0.0 (fixed)	–	–	–	–
	s_2,2_	0.01	0.009	0.129	−0.113	1.054	100

**Table 4.** Results from simulation scenarios 7 and 8. Transmission rate ratio and sampling proportions were jointly estimated for transmission trees belonging to the same tree class. For Bayesian inference, r_λ,1_ was fixed to its true value (r_λ,1_ = 1.0). Out of 100 simulation replicates, for scenario 7 in total 89 replicates and for scenario 8 in total 99 replicates yielded ESS > 200 for all estimated parameters.

Scenario	Parameter	Truth	Median	Relative error	Relative bias	Relative HPD width	95% HPD accuracy
7	R_base, t1_	3.0	2.876	0.078	−0.041	0.415	97
	R_base, t2_	2.3	2.338	0.034	0.017	0.175	98
	r_λ,1_	1.0	1.0 (fixed)	–	–	–	–
	r_λ,2_	1.666	1.626	0.069	−0.024	0.366	96
	s_1,1_	0.0	0.0 (fixed)	–	–	–	–
	s_1,2_	0.01	0.010	0.123	0.010	0.918	100
	s_2,1_	0.0	0.0 (fixed)	–	–	–	–
	s_2,2_	0.01	0.010	0.111	−0.017	1.067	100
8	R_base, t1_	1.17	1.260	0.094	0.077	0.343	87
	R_base, t2_	0.9	1.008	0.120	0.120	0.084	0
	r_λ,1_	1.0	1.0 (fixed)	–	–	–	–
	r_λ,2_	1.6	1.402	0.124	−0.124	0.188	29
	s_1,1_	0.0	0.0 (fixed)	–	–	–	–
	s_1,2_	0.01	0.012	0.178	0.152	0.692	93
	s_2,1_	0.0	0.0 (fixed)	–	–	–	–
	s_2,2_	0.01	0.010	0.137	−0.01	0.907	100

For scenarios 1–3, all the parameters inferred were recovered reliably (Table 2). In each case, the median estimates for ${R_{base}}$, ${r_\lambda }$, and *s* were close to the true values and the relative HPDI widths were narrow, implying good accuracy and precision. In each case, the recovered median values for ${R_{base}}$ (3.01–3.06) were marginally higher than the true value, whereas the median estimates for the transmission rate ratio ${r_{\lambda ,high}}$ were somewhat lower (1.57–1.64). Nevertheless, high accuracy was achieved for each of the parameters under scrutiny with values ranking from 92 to 100 per cent.

In scenario 4, transmission trees with ${N_{tips}} = $50 were simulated whereas scenario 5 in addition contained trees with 10 and 20 tips and scenario 6 further included two-sequence transmission trees. The number of tips had a notable impact: for scenarios 4 and 5, ${R_{base}}\,$was estimated with high 95 per cent HPD accuracy (over 90 per cent for both) and median estimates were close to the true value (${R_{base}} = $ 2.95 for both) (Table 3, Supplementary Figure S1). Conversely, when two-sequence trees are included, ${R_{base}}\,$was underestimated, and the accuracy decreased considerably compared to the previous simulations (${R_{base}} = $ 2.69, 95 per cent HPD accuracy of 44 per cent) (Table 2, Supplementary Figure S1). For scenarios 4–6, the transmission rate ratio parameter ${r_{\lambda ,2}}$ was inferred with relatively high accuracy (95 per cent HPD accuracy of 91–97 per cent), even though median values were slightly underestimated.

In scenario 7, a decline in ${R_{base}}\,$was recovered with high confidence in both time intervals as the mean estimates were close to the truth and the 95 per cent HPD accuracy between 97 per cent and 98 per cent (Table 4, Supplementary Figure S2). Moreover, ${r_{\lambda ,2}}$ was recovered well. Contrarily, in scenario 8, ${R_{base}}\,$was overestimated, yielding median values of ${R_{base,t1}} = $ 1.26 and ${R_{base,t2}} = $ 1.0 (Table 4). Whereas for the majority of simulation replicates, the posterior distributions of ${R_{base,t1}}$ included the true value, for ${R_{base,t2}}$ relative HDPIs were remarkably narrow (width of 0.083). As a result, the posterior distributions were constantly above the true value (Supplementary Figure S3). Consequently, estimates for ${r_{\lambda ,2}}$ were notably lower than the true value with a median of ${r_{\lambda ,2}} = $ 1.40 and relative bias of −0.12. Due to the overestimation and narrow relative HPDI width, the accuracy for ${R_{base,t2}}$ is zero, whereas for ${r_{\lambda ,2}}$ an accuracy of 29 per cent is achieved.

In scenarios 9 and 10, we assessed the sensitivity of the model towards variation in transmission rates within a tree class: trees representing the ${r_{\lambda ,low}}$ class were simulated by setting ${r_{\lambda ,low}} = $ 0.8–1.2 and trees belonging to the ${r_{\lambda ,high}}$ by setting ${r_{\lambda ,high}} = $ 1.4–1.8. Transmission rates were inferred independently (scenario 9) or jointly for trees belonging to the same tree class (scenario 10). For both scenarios, ${R_{base}}$ and the associated scaling factors were inferred with high accuracy (95 per cent HPD accuracy of 91–98 per cent) (Table 5 and Supplementary Figure S4). Scenario 10 shows that in the joint analysis the inferred value corresponds to the mean of the combined trees, with narrow HPDIs.

**Table 5.** Results from simulation scenarios 9 and 10. For Bayesian inference, scenario 9, r_λ,3_ was fixed to its true value (r_λ,3_ = 1.0). Correspondingly, for scenario 10 r_λ,1_ was fixed to its true value (r_λ,1_ = 1.0). For both scenarios, all simulation replicates yielded ESS > 200 for all estimated parameters.

Scenario	Parameter	Truth	Median	Relative error	Relative bias	Relative HPD width	95% HPD accuracy
9	R_base_	3.0	3.065	0.078	0.022	0.388	95
	r_λ,1_	0.8	0.802	0.108	0.002	0.515	94
	r_λ,2_	0.9	0.903	0.115	0.003	0.532	96
	r_λ,3_	1.0	1.0 (fixed)	–	–	–	–
	r_λ,4_	1.1	1.084	0.110	−0.015	0.551	91
	r_λ,5_	1.2	1.217	0.115	0.014	0.587	96
	r_λ,6_	1.4	1.399	0.108	−0.001	0.600	95
	r_λ,7_	1.5	1.513	0.109	0.008	0.613	98
	r_λ,8_	1.6	1.605	0.117	0.003	0.619	96
	r_λ,9_	1.7	1.669	0.109	−0.018	0.613	95
	r_λ,10_	1.8	1.785	0.126	−0.008	0.627	97
	s_1_	0.01	0.01	0.076	−0.009	1.134	100
	s_2_	0.01	0.01	0.074	−0.022	1.136	100
	s_3_	0.01	0.01	0.074	−0.030	1.137	100
	s_4_	0.01	0.01	0.067	−0.031	1.143	100
	s_5_	0.01	0.01	0.074	−0.035	1.144	100
	s_6_	0.01	0.01	0.058	−0.024	1.160	100
	s_7_	0.01	0.01	0.067	−0.019	1.166	100
	s_8_	0.01	0.01	0.053	−0.025	1.165	100
	s_9_	0.01	0.01	0.056	−0.026	1.165	100
	s_10_	0.01	0.01	0.053	−0.027	1.166	100
10	R_base_	3.0	2.924	0.038	−0.025	0.184	94
	r_λ,1_	1.0	1.0 (fixed)	–	–	–	–
	r_λ,2_	1.6	1.636	0.055	0.022	0.302	96
	s_1_	0.01	0.010	0.131	0.019	1.010	100
	s_2_	0.01	0.010	0.107	−0.003	1.073	100

### SARS-CoV-2 data analysis

**Figure 1.**
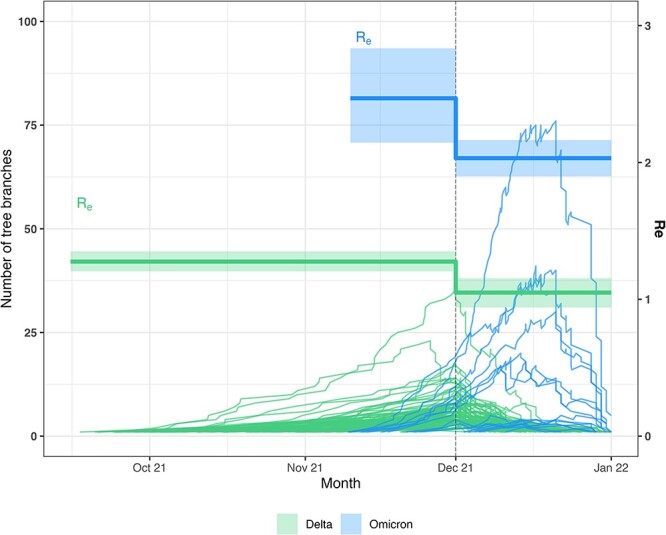
Lineage-through-time plot (LTT, left Y-axis) of inferred summary trees and effective reproductive number (${R_e}$, right Y-axis) through time. Each line represents a Delta-associated (green) or Omicron-associated (blue) cluster. The vertical dotted line shows the time point at which the sampling proportion and baseline reproductive number were allowed to change. Bold ${R_e}$ lines correspond to the median estimate, the shaded area to the 95 per cent HPDIs. The lines for the Omicron ${R_e}$ estimate are calculated by multiplying the transmission rate ratio with ${R_{base}}$, as the variants share the same rate to become non-infectious.

**Figure 2.**
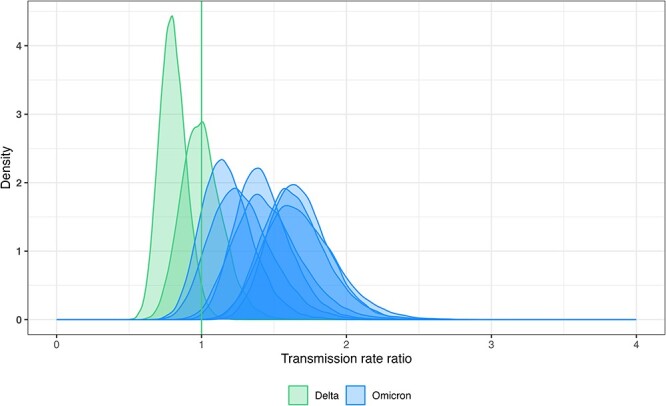
Sampled posterior distributions for cluster-specific transmission rate ratios for Delta- (green) and Omicron-associated clusters (blue). The vertical line belongs to the Delta cluster for which the transmission rate ratio was fixed to one.

We applied the presented method to publicly available SARS-CoV-2 genomes from the federal state of Berlin, Germany, sampled during December 2021, with the aim of describing the local transmission advantage of the Omicron over the Delta variant. For this, we first inferred one transmission rate ratio for all Omicron- relative to all Delta-associated clusters and second individual ratios for all clusters. The main results are illustrated in Figs 1 and 2. The Delta-associated clusters reach further back in time than those comprising Omicron sequences. This is in line with the much more recent emergence of the Omicron variant and the long circulation of Delta previously in the area. It is also striking that most Delta clusters start to decline before the sampled period, i.e. most branching events in the tree are reconstructed to have happened before December. In contrast, the Omicron-associated clusters show decreasing LTT plots only close to the end of the sampled period. As the reproductive number is informed by the timing of the branching events in the trees, we see this trend reflected therein. The inferred Delta-associated baseline reproductive number changes from above one (median 1.28, 95 per cent HPDI [1.21,1.35]) in the first interval, to a median estimate of 1.05 (95 per cent HPDI [0.94,1.15]) in the second interval. This indicates a growing epidemic in the first and a declining one in the second interval. We thus reconstruct an impactful change in the transmission dynamics of Delta around the start of the sampling period, namely the stop of an increase in infections. The relative difference of the reproductive number of Omicron-associated clusters is given by the inferred transmission rate ratio. As illustrated in Fig. 1, this lifts the reproductive number of Omicron in our sample to values above one in both intervals. Other than Delta, Omicron cases thus do not stagnate at the beginning of December, but still increase. With a median transmission rate ratio of 1.93 (95 per cent HPDI [1.72,2.17]), the transmission advantage of Omicron over Delta in Berlin in December 2021 is almost two-fold.

To evaluate the variation in the transmission rate between clusters of the same variant, we conducted a second analysis in which we inferred individual transmission rate ratios for all clusters with at least 20 sequences. The posterior densities for the transmission rate ratios are shown in Fig. 2. We reconstruct a clear trend from Delta to Omicron in the median estimates of the transmission rate ratios, however, with overlapping HPDIs for all clusters. For Omicron-associated clusters, we see a pronounced heterogeneity in the cluster-specific transmission rate ratio relative to Delta, as median estimates reach from 1.18 up to 1.69 (95 per cent HPDIs [0.85,1.54] and [1.22,2.18]). For Delta, less heterogeneity is observed, likely due to the small number of clusters.

In Supplementary text S5, we discuss multiple analyses testing the sensitivity of our results to changes in the data and model setup. These analyses support the overall robustness of our results (Supplementary Tables S3 and S4, as well as Supplementary Figure S5).

## Discussion

In this paper, we introduce and test BDSKYλ, an extension to the Bayesian birth–death skyline model that allows the inference of relative transmission rates from a set of trees. By allowing variation in the transmissibility, we are able to distinguish a potential transmission advantage of one or multiple cluster(s) over the other(s). This property is of interest, for instance, when considering the COVID-19 pandemic which has so far been characterized through the continuous displacement of the dominantly circulating variant by another due to e.g. higher transmissibility or immune evasion (Martin et al. 2022). For SARS-CoV-2, modelling a scaling factor for the transmission rate, also better resembles the biological transmission mechanism, which is, in principle, unchanged between variants. Instead, few genetic differences lead to an enhancement of the transmission process through, for example, increased ACE2 receptor binding affinity of N501Y lineages (Martin et al. 2021).

We evaluated the validity of the BDSKYλ approach with simulation studies under several scenarios. We particularly tested the model performance under different transmission rates, sampling proportions, and sample sizes as well as regarding the joint analysis of multiple trees with linked epidemiological parameters. Such joint inference has previously been used for the analysis of different genomic datasets within the BDSKY framework (Müller et al. 2020; Nadeau et al. 2023) (Müller et al. 2020; Nadeau et al. 2023), but so far lacks a published validity study. We showed that for the majority of cases the model recovered reliably both epidemiological parameters investigated. Assuming a fixed rate to become non-infectious, high accuracy was obtained consistently and transmission rates are estimated with good precision, in most instances. Moreover, our simulation results demonstrated that the model can precisely infer shared epidemiological parameters for transmission trees with similar transmission properties. It also highlights how linking the transmission rate ratios between clusters can increase the statistical power by narrowing the HPDIs for the estimated transmission advantage.

Although BDSKYλ re-estimates epidemiological parameters reliably for most scenarios, the model tends to struggle in two cases: (i) when individual transmission trees contain only few samples and (ii) when the true reproductive number is below one. For simulated transmission trees with at least 10 samples, transmission rates are inferred with high precision. The performance of the model decreases drastically when two-sequence transmission trees are included. We hypothesize that sensitivity to small transmission trees is a more general complication for birth–death models; as for the small clusters, the impact of the stochasticity of the process increases. We therefore opted to exclude clusters of less than four sequences from analyses with empirical data. Furthermore, when applying BDSKYλ to simulation scenarios with ${R_e}$ values below one (scenario 8), the model heavily overestimates the reproductive number, yielding exclusively posterior estimates above 1.0. By introducing low transmission rates, one tends to select a biased set of simulated transmission trees as we exclude phylogenies that die out before the requested number of tips have been sampled, as described previously (Boskova, Bonhoeffer, and Stadler 2014).

With the analysis of SARS-CoV-2 genomes sampled in Berlin in December 2021, we present a quantification of the transmission advantage of Omicron over Delta in Germany purely based on molecular surveillance data. These results broaden the understanding of the transmission dynamics of SARS-CoV-2 in a crucial period in Germany characterized by evolutionary changes in viral transmissibility and public intervention measures of pharmaceutical and non-pharmaceutical nature. With an overall transmission rate ratio of around two, the inferred transmission advantage of Omicron is smaller than previously determined from genomic data in other regions (Maier et al. 2023), e.g. early after its emergence in South Africa or in Denmark (Ito, Piantham, and Nishiura 2022; Viana et al. 2022), but in line with estimates from variant screening tests in France (Sofonea et al. 2022). Variations between areas are likely caused by demographic differences between the population in which the disease spread, similar to the observed variation between clusters. In Berlin and in Germany, COVID-19 case counts increased drastically in autumn 2021 in a wave of new Delta infections, which lead to the reintroduction of multiple non-pharmaceutical interventions during November 2021. These NPIs stopped the increase of Delta infections in the area (https://www.rki.de/DE/Content/InfAZ/N/Neuartiges_Coronavirus/Situationsberichte/Wochenbericht/Wochenberichte_Tab.html, last visited 11 July 2023), which is reflected in our estimates of the baseline reproductive number of around one in the sampled interval (see Fig. 1). They were, however, not successful in halting the fast spread of the Omicron variant, as the rise of case numbers starting mid-December 2021 shows. Through our estimates of the baseline reproductive number in the second interval and transmission rate ratio for Omicron we quantify this effect. As we only infer one scaling factor for the baseline transmission rate, we do not quantify if the transmission advantage is caused through intrinsic or extrinsic changes of the viral transmissibility. However, through the inference of cluster-specific transmission rate ratios, we point out how variable this advantage is between clusters. Similar observations have been made for SARS-CoV-2, for example for several VOCs in the UK (Volz 2023). This highlights the impact of demographic factors on the population-level transmissibility and the need to combine multiple clusters, representative of the whole population, for the estimation of overall transmission differences.

We would, however, like to highlight some limiting factors of our analyses: First, we used purely genetic distance to approximate transmission chains within the considered area. Counts of genetic differences, acquired over a short period of time, do not necessarily hold all the information required to distinguish a common local transmission history. Second, like all general birth–death-sampling models, we assumed an infinite susceptible population and therefore did not account for potential differences in the susceptible population size between variants or partial depletion of the pool of susceptible. In Berlin in December 2021, the background immunity against Delta was already relatively high (COVID-19-Impfungen in Deutschland, https://zenodo.org/records/6942355, last visited 21 October 2023), while the Omicron variant has been shown to have strong invasive properties from immunity against previous SARS-CoV-2 variants (Markov et al. 2023). This difference in the size of the susceptible population could potentially impact our results. We, third, inferred a relatively high sampling proportion of Delta sequences in the considered time period and area. This could be due to the genetic sample not being fully representative of the entire viral diversity circulating at the time in the area, for example due to the sequence data set focusing more on the city Berlin than the state. Alternatively, it could reflect a higher frequency of non-VOC lineages than identified by variant-specific PCR tests. Another explanation could be a drastically changing sampling rate throughout the month (Volz and Frost 2014). Lastly, due to convergence issues we opted to exclude data, namely sequences that were assigned to clusters with less than four sequences. As trees of only very few sequences can, in general, be statistically problematic due to their small sample size, we recommend being careful when jointly analysing many trees like this. In particular, exclusion thresholds and their impact on the inferred transmission dynamics should be evaluated independently for each dataset, since the information content carried by small clusters will, among other things, depend on the genetic diversity in the data and the setup of the analysis. To address these limitations, we provide several sensitivity analyses, demonstrating the robustness of our transmission rate ratio estimates. These also show a pronounced decrease in the estimated sampling proportion for different cluster size exclusion thresholds, supporting the robust parameter estimation.

As the original birth–death skyline model has been an advantageous tool when describing past changes in a pathogen population, the BDSKYλ model, presented here, introduces further flexibility for studying epidemiological dynamics. The model provides a framework to infer a baseline reproductive number and scaling factors informed by multiple transmission trees at once. This allows the joint analysis of multiple independent clusters without running a computationally more complex multi-type birth–death model. Since the method scales comparably to independent BDSKY analyses on the considered data, it can still be well applied to datasets of few thousand sequences in total. It thus facilitates the analysis of a viral population encompassing multiple transmission clusters with heterogeneous transmission patterns, which is not only relevant for SARS-CoV-2 but also for several other measurably evolving pathogens such as HIV and influenza. Extensions that allow relative differences in other model parameters, i.e. the death or sampling rate, would be straightforward to implement.

## Supplementary Material

vead070_Supp

## Data Availability

No new data were generated or analysed in support of this research. The data underlying this article are available in GISAID, at gisaid.org under the accession IDs listed in Supplementary Table S2. Example xml files and R scripts, for evaluation and visualisation, are provided on GitHub, at https://github.com/tidelab/BDSKYlambda_manuscript.
