# Supplementary material for: Reconstructing relative transmission rates in Bayesian phylodynamics: Two-fold transmission advantage of Omicron in Berlin, Germany during December 2021
Source: Virus Evol. 2023 Nov 29;9(2):vead070. doi: 10.1093/ve/vead070 (PMC10725310; doi:10.1093/ve/vead070)
Supplement: vead070_Supp [file vead070_supp.zip › vead070_Supp/suppl_data/Weber_Oeversti_Kuehnert_SupplementaryInformation_VEVOLU-2023-127_2023-10-30.pdf]

Reconstructing relative transmission rates in Bayesian phylodynamics: Two-fold advantage of Omicron in Berlin, Germany during December 2021

Ariane Weber, Sanni Översti, Denise Kühnert

Supplementary Material

Content

Supplementary text S1. Definitions of technical terms used in this study.
Supplementary text S2. Detailed description of BDSKYλ method.
Supplementary text S3. Detailed description of simulation studies.
Supplementary text S4. Calculation of genetic cluster threshold.
Supplementary text S5. Sensitivity analyses for the SARS-CoV-2 data set.

Supplementary table S1. Summary of simulation scenarios used in this study.
Supplementary table S2. GISAID acknowledgements.
Supplementary table S3. Parameter estimates for the analysis presented in the main text and the three sensitivity analyses.
Supplementary table S4. Parameter estimates for the analysis presented in the main text and two other cluster size inclusion thresholds.

Supplementary figure S1. Estimated posterior distributions for R_{base} and $r_{\lambda,high}$ for simulation scenarios 4–6.
Supplementary figure S2. Estimated posterior distributions for $R_{base,t1}$, $R_{base,t2}$ and $r_{\lambda,high}$ for simulation scenario 7.
Supplementary figure S3. Estimated posterior distributions for $R_{base,t1}$, $R_{base,t2}$ and $r_{\lambda,high}$ for simulation scenario 8.
Supplementary figure S4. Comparison of estimated posterior distributions for $r_{\lambda,low}$ and $r_{\lambda,high}$ between simulation scenarios 9 and 10.
Supplementary figure S5. Impact of cluster size distribution on SARS-CoV-2 data analysis.

Supplementary text S1. Definitions of technical terms used in this study.
Throughout this publication, we define the listed terms in the following way:

Transmission cluster = Group of infected individuals from a panmictic population. Sharing homogeneous transmission dynamics starting from a cluster-specific index patient.

Transmission tree = Binary tree representation of the time course of a transmission process. Throughout this paper we assume a transmission tree to arise from a birth-death process which allows incomplete sampling. In this tree the internal nodes correspond to transmission events and tips correspond to sampling events.

Phylogenetic tree = Tree reconstructed from (viral) genetic sequences.

Genetic cluster = A group of pathogen sequences selected based on the genetic differences. In this paper we approximate the transmission clusters through the genetic clusters.

Supplementary text S2. Detailed description of the BDSKYλ method.
The birth-death skyline model describes the stochastic changes in the size of a population of infected individuals through time. Starting at time $\tau = 0$ with one infected individual, size increments happen with transmission rate λ while population size decrements with recovery rate μ and sampling rate ψ. All three rates are piecewise constant with changes occurring at times t. Additionally, another sampling scheme is allowed for which at times $t_{1,\ldots,m}$ each individual is sampled with rate ρ. From these five parameters, the probability density of a sampled tree $f(T|\lambda,\mu,\psi,\rho,t)$ is defined in Theorem 1 in (Stadler et al. 2013). Together with the likelihood $P(D|T,v,S)$ and marginal likelihood $f(D)$ this yields the full posterior density of a tree T and its defining parameters

$$f(T,v,S,\lambda,\mu,\psi,\rho,t|D) = \frac{P(D|T,v,S)f(T|\lambda,\mu,\psi,\rho,t)f(v,S,\lambda,\mu,\psi,\rho,t)}{f(D)}.$$

Here D denotes data, i.e. the multiple sequence alignment (MSA), v the substitution rate and S substitution model specific parameters. The model can be re-parameterised to directly quantify the reproductive number R, the inverse of the duration of infectiousness δ (rate to become non-infectious) and the sampling probability s:

$$R = \frac{\lambda}{\delta},\ \ \delta = \mu + \psi, \text{ and }\ s = \frac{\psi}{\mu+\psi}.$$

If we consider multiple trees $T = \{T_1,\ldots,T_n\}$ that are independent realizations of the same stochastic birth-death-sampling process with parameters Θ, these can be reconstructed simultaneously through a joint posterior distribution from their MSAs $D = \{D_1,\ldots,D_n\}$:

$$f(T,v,S,\Theta|D) = \frac{f(v,S,\Theta)\prod_{i=1}^{n} f(T_i|\Theta)P(D_i|T_i,v,S)}{f(D)}$$

To allow for relative differences in the transmission rate between jointly inferred trees, we model for each tree a scaling factor r_λ of the baseline transmission rate λ_{base}. The corresponding posterior probability distribution then takes the form

$$f(T,v,S,\Theta|D) = \frac{f(v,S,\lambda_{base},\mu,\psi,\rho,t)\prod_{i=1}^{n} f(T_i|\lambda_{base}r_{\lambda,i},\mu,\psi,\rho,t)f(r_{\lambda,i})P(D_i|T_i,v,S)}{f(D)}.$$

The (absolute) baseline transmission rate corresponds to one predefined transmission cluster k through specifying $r_{\lambda,k} = 1$. If we assume a fixed, shared rate to become non-infectious, the inferred transmission rate ratio $r_{\lambda,i}$ will also quantify the scaling factor for the baseline reproductive number R_{base} in the epidemiological parameterisation of the model.

Supplementary text S3. Detailed description of simulation studies.
For all simulation scenarios we assumed a rate to become non-infectious of $\delta = 36.5$ years^{-1} (Byrne et al. 2020; Lam and Duchene 2021; Nadeau et al. 2021; NIID 2022; Siedner et al. 2022) that corresponds to an average infectious period of ten days. In simulation scenarios 1–7 the values for R_{base} and r_{λ} were established on the basis of previously published estimates: to determine R_{base} we used basic reproductive number estimates of the ancestral SARS-CoV-2 strain presented in (Liu et al. 2020). They found a mean basic reproductive number of 3.28 and median of 2.79, thus we set $R_{base} = 3.0$. For the transmission rate ratio parameter, we applied two classes: $r_{\lambda,low}$ and $r_{\lambda,high}$. For the class $r_{\lambda,low}$ we assumed the transmission rate to correspond to R_{base}, implying that $r_{\lambda,low} = 1.0$. The transmission rate for the category $r_{\lambda,high}$ was determined based on R estimates of the Delta variant. The meta-analysis presented in (Liu and Rocklöv 2021) obtained a mean estimate of 5.08 for Delta, therefore we assumed $r_{\lambda,high} = 1.666$. Furthermore, in scenario 7 we simulated a piecewise decline in the transmission rate by setting the effective reproductive number in the first interval to $R_{base,t1} = 3.0$ and in the second interval to $R_{base,t2} = 2.3$ (retaining $r_{\lambda,low}$ =1.0 and $r_{\lambda,high} = 1.666$). The change in R_{base} was set to occur at $t = 0.058$y which corresponds to approximately 21 days. In scenario 8 we aimed to test the performance of the model when the epidemic is considered to grow in the first time interval whereas in the second interval it is declining ($R_{base,t1} > 1$ and $R_{base,t2} < 1$). Again, we assumed two transmission rate classes, $r_{\lambda,low}$ and $r_{\lambda,high}$, and set the transmission rates so that $R_{base,t1} = 1.17$, $R_{base,t2} = 0.9$, $r_{\lambda,low} = 1.0$ and $r_{\lambda,high} = 1.6$. The change in R_{base} was set to occur at $t = 0.15$y, which corresponds to approximately 55 days. In scenarios 9 and 10, we further evaluated the model's performance under a situation where trees belonging to the same transmission rate ratio class exhibit small-scale heterogeneity in the transmission rates. For this, trees representing lower transmission rate were simulated with $r_{\lambda,low} = 0.8$–1.2 and trees belonging to the higher transmission rate with $r_{\lambda,high} = 1.4$–1.8. For both scenarios we assumed R_{base} =3.0.

The number of simulated transmission trees varied between scenarios: in scenarios 1–3 five trees were simulated whereas for the rest of the scenarios the number of simulated transmission trees varied from six to 36 (for details, see supplementary text S1 and supplementary table S1). In scenarios 2–8 for particular transmission trees the onset of the sampling process was prolonged, meaning that within genealogy sampling and transmission processes did not start simultaneously. The sampling process was delayed to reflect a real-life situation where a detection lag of a transmission lineage has been reported to be on average 14.13 ± 5.61 days (du Plessis et al. 2021).

For Bayesian inference a strict molecular clock model was used with a fixed substitution rate of 0.0008 substitutions/site/year (Ghafari et al. 2020). Albeit several different rate estimates have been proposed for SARS-CoV-2, rates obtained with phylodynamic approaches range from 0.00058 to 0.0013 substitutions/site/year (see Table 1 in (Attwood et al. 2022)). Moreover, all tree classes share the same fixed rate, since it has been shown that notable acceleration of SARS-CoV-2 substitution rates occurred particularly in the basal clades leading to VOCs while molecular rate estimates between individual clades are highly overlapping (Tay et al. 2022).

Correspondingly, for Bayesian inference we fixed the rate to become non-infectious to its true value ($\delta = 36.5$ years^{-1}). As a prior distribution for R_{base} we used a Lognormal(0.0, 1.25) distribution in all scenarios. In scenarios 1–3 and 9 the transmission rate ratios were estimated independently for each transmission tree. In scenarios 4–8 and 10 the transmission rate ratios were inferred jointly for transmission trees belonging to the same tree class, i.e. we inferred one r_{λ} estimate for all transmission trees simulated with lower transmission rate ratio ($r_{\lambda,low}$) and one r_{λ} for all trees simulated with higher transmission rate ratio ($r_{\lambda,high}$). In all cases we assumed a lognormal (0.0, 1.0) prior distribution.

In scenarios 1–3 and 9 the sampling proportions were estimated independently for each transmission tree. For trees for which the sampling rate equalled 1% we used a Beta(10, 990) prior distribution and for trees for which the sampling rate was 0.1% we assumed beta(1, 99). Similarly to the transmission rate ratios, in scenarios 4–8 and 10 sampling proportions were inferred jointly for transmission trees

belonging to the same tree class with Beta(10, 990) as a prior. In scenarios 2–8 the sampling delays introduced were inferred by setting a sampling rate change time to $t = 0.019$ or $t = 0.038$ that corresponds to 7 and 14 days, respectively. In scenarios 7 and 8, the piecewise changes in R_{base} were similarly inferred by setting a transmission rate change time to $t = 0.058$ or $t = 0.15$ corresponding to approximately 21 and 55 days, respectively.

Supplementary text S4. Calculation of genetic cluster threshold.

For the empirical SARS-CoV-2 data from Berlin (see Methods) genetic clusters were defined with ClusterPicker1.2.5 (Ragonnet-Cronin et al. 2013). Clusters were identified by setting a pairwise genetic distance threshold, given in percentages. The maximum genetic distance allowed within a cluster was determined based on the substitution rate estimate obtained in Tay et al. 2022. Briefly, Tay et al. 2022 evaluated if emergence of variants of concern (VOC) can be assigned to changes in substitution rate over time. By fitting various molecular clock models into a data set containing sequences from Alpha, Beta, Gamma, and Delta lineages, authors concluded that emergence of these four lineages can be attributed to the episodic increase in the substitution rate of the VOC stem branches. These so-called ‘foreground’ branches had up to 4-fold increase in the substitution rate compared to the ‘background’ branches, the latter including also all four VOC clades (see Tay et al. 2022 supplementary figure S1). The estimated foreground rate was 2.45 x 10^{-3} subst./site/year (95% CI: 1.15 – 4.72 x 10^{-3}) whereas the estimated background rate was 0.58 x 10^{-3} subst./site/year (95% CI: 0.51 – 0.65 x 10^{-3}). We chose to use the mean of substitution rate of the foreground branches as an upper limit for the pairwise genetic distance when defining genetic clusters for the SARS-CoV-2 data sampled from Berlin within one month. Assuming genome size of 29903 bp, a rate of 2.45 x 10^{-3} (8.71 x 10^{-4}) subst./site/year would convert into approximately 73.26 substitutions (26.02) per genome per year leading to around 6.11 (2.2) substitutions per genome per month. Accordingly, the maximum expected number of substitutions between two sequences within one month would be 12.22 (4.4) meaning a 0.041% (0.015%) difference. Comparable maximum pairwise distance of 0.0004 has been used previously in (Seemann et al. 2020) for SARS-CoV-2 cluster identification. We used the same genetic distance threshold for Omicron as for Delta. Whereas some studies have reported for Omicron higher substitution rates over Delta, others have presented contradictory results (for discussion see (Markov et al. 2023)). As no systematic indication of distinct evolutionary rates for Omicron and Delta could be found, we consider the assumption of equal rates to be less problematic than, for example, the assumption of higher rates for Omicron. The size distribution of the resulting clusters is shown in Supplementary Figure S5. Similar distributions have also been described for SARS-CoV-2, e.g. for importation lineages identified through phylogeographic analyses in the UK (du Plessis et al. 2021) and New Zealand (Geoghegan et al. 2020).

Supplementary text S5. Sensitivity analyses for the SARS-CoV-2 data set.

To evaluate the impact of the sampling scheme and prior distributions on the results, we performed sensitivity analyses regarding the sampling scheme, duration of infectiousness, sampling proportion and minimum cluster size. To check for potential biases arising from non-random sampling, sequences in the initial data set were removed if they did not contain the metadata label ‘random sampling’ under ‘sampling scheme’ (e.g. sequences labelled ‘suspect sampling’). All subsequent analyses were performed on the ‘random sampling’ data set, comprising 808 sequences in the final BDSKYλ analysis. Following previous publications finding a slightly reduced duration of PCR-test positivity of Omicron infections, in a second sensitivity setup, a separate become non-infectious rate of 40.5 years^{-1} was modelled for Omicron-associated clusters. Thirdly, the impact of the absolute value of the inferred sampling proportion and of the relative difference between variants was evaluated by running an alternative analysis in which the sampling proportion for both variants was fixed to 0.04.

While we infer a median sampling proportion of 5.7% for Omicron sequences, close to the mean of the prior distribution, we find the Delta estimates to be much higher with a median of 11.7%. The inferred transmission rate ratio, however, is robust against changes in the sampling proportion, as demonstrated by overlapping HPDIs with the analysis in which the sampling proportion for both variants is fixed to 4%. Comparing the inferred transmission rates, the same holds for the former two sensitivity analyses

(see supplementary table S4). Lastly, we tested the impact of the smallest included cluster size on the inferred transmission rate ratio as we found the size distribution of the inferred genetic distance clusters to exponentially decline, i.e. we see many very small and very few big clusters (see supplementary figure S5). We therefore ran the same analysis setup for datasets in which we included only clusters with i) at least four sequences, ii) at least ten sequences and iii) at least twenty sequences. We also tried analyses including all clusters – these, however, did not converge, i.e. not all inferred parameters reached ESS values over 200, namely tree likelihood and tree prior for some of the smallest clusters. The results from these analyses show the transmission rate ratio to be slightly sensitive to excluding smaller clusters since the median estimates differ between the three analyses. However, the inferred HPDIs overlap significantly, supporting our results presented in the main text for analysis i).

Supplementary table S1. Summary of simulation scenarios used in this study. For all scenarios, except for scenario 1, all transmission trees were simulated by assuming a sampling rate $\psi = 0.365$ and a recovery rate of $\mu = 36.135$. For scenario 1 transmission trees 1–4 were simulated with $\psi = 0.365$ and $\mu = 36.135$, whereas for transmission tree 5 a sampling rate of $\psi = 0.0365$ and recovery rate $\mu = 36.4635$ were used. For the Bayesian inference for all the scenarios we fixed rate to become non-infectious to $\delta = 36.5$ and substitution rate to 0.0008 substitutions/site/year. Additionally, for all scenarios Lognormal(0.0, 1.25) and Lognormal(0.0, 1.0) were used as prior distributions for R_{base} and for r_λ, respectively. Unless stated otherwise, for sampling proportion as a prior distribution Beta(10, 990) was used.

**Scenario**	**Simulation settings**			**Bayesian inference**	
	**Transmission parameters**	**Transmission trees**	**Delay in the sampling process**	**Sampling proportion (*s*)**	**Transmission rate ratio (r_λ)**
1	$R_{base} = 3.0$<br>$r_{\lambda,low} = 1.0$<br>$r_{\lambda,high} = 1.666$	1–4: $r_{\lambda,low}$ and $N_{tips} = 25$<br>5: $r_{\lambda,high}$ and $N_{tips} = 250$	1–5: none	Inferred independently for each transmission tree.<br>Prior for transmission tree 5 Beta(1,99).	Inferred independently for each transmission tree.
2	$R_{base} = 3.0$<br>$r_{\lambda,low} = 1.0$<br>$r_{\lambda,high} = 1.666$	1–4: $r_{\lambda,low}$ and $N_{tips} = 25$<br>5: $r_{\lambda,high}$ and $N_{tips} = 25$	1–4: none<br>5: 14 days	Inferred independently for each transmission tree.<br>SamplingRateChangeTime for $r_{\lambda,high}$ (t = 0.038y).	Inferred independently for each transmission tree.
3	$R_{base} = 3.0$<br>$r_{\lambda,low} = 1.0$<br>$r_{\lambda,high} = 1.666$	1–4: $r_{\lambda,low}$ and $N_{tips} = 100$<br>5: $r_{\lambda,high}$ and $N_{tips} = 100$	1–4: none<br>5: 14 days	Inferred independently for each transmission tree.<br>SamplingRateChangeTime for $r_{\lambda,high}$ (t = 0.038y).	Inferred independently for each transmission tree.
4	$R_{base} = 3.0$<br>$r_{\lambda,low} = 1.0$<br>$r_{\lambda,high} = 1.666$	1–3: $r_{\lambda,low}$ and $N_{tips} = 50$<br>4–6: $r_{\lambda,high}$ and $N_{tips} = 50$	1–3: none<br>4–6: 14 days	Inferred jointly for transmission trees with $r_{\lambda,low}$ (i.e. trees 1–3) as well as jointly for transmission trees with $r_{\lambda,high}$ (i.e. trees 4–6).<br>SamplingRateChangeTime set for $r_{\lambda,high}$ (t = 0.038y).	Inferred jointly for transmission trees with $r_{\lambda,low}$ (i.e. trees 1–3) as well as jointly for transmission trees with $r_{\lambda,high}$ (i.e. trees 4–6).
5	$R_{base} = 3.0$<br>$r_{\lambda,low} = 1.0$<br>$r_{\lambda,high} = 1.666$	1: $r_{\lambda,low}$ and $N_{tips} = 10$<br>2: $r_{\lambda,low}$ and $N_{tips} = 20$<br>3: $r_{\lambda,low}$ and $N_{tips} = 50$<br>4: $r_{\lambda,high}$ and $N_{tips} = 10$<br>5: $r_{\lambda,high}$ and $N_{tips} = 20$<br>6: $r_{\lambda,high}$ and $N_{tips} = 50$	1–3: none<br>4–6: 14 days	Inferred jointly for transmission trees with $r_{\lambda,low}$ (i.e. trees 1–3) as well as jointly for transmission trees with $r_{\lambda,high}$ (i.e. trees 4–6).<br>SamplingRateChangeTime set for $r_{\lambda,high}$ (t = 0.038y).	Inferred jointly for transmission trees with $r_{\lambda,low}$ (i.e. trees 1–3) as well as jointly for transmission trees with $r_{\lambda,high}$ (i.e. trees 4–6).

6	$R_{base} = 3.0$<br>$r_{\lambda,low} = 1.0$<br>$r_{\lambda,high} = 1.666$	1–3: $r_{\lambda,low}$ and $N_{tips} = 50$<br>4–6: $r_{\lambda,high}$ and $N_{tips} = 50$<br>7–11: $r_{\lambda,low}$ and $N_{tips} = 2$<br>12–16: $r_{\lambda,low}$ and $N_{tips} = 2$<br>17–21: $r_{\lambda,low}$ and $N_{tips} = 2$<br>22–26: $r_{\lambda,high}$ and $N_{tips} = 2$<br>27–31: $r_{\lambda,high}$ and $N_{tips} = 2$<br>32–36: $r_{\lambda,high}$ and $N_{tips} = 2$	1–3: none<br>4–6: 14 days<br>7–11: none<br>12–16: 7 days<br>17–21: 14 days<br>22–26: none<br>27–31: 7 days<br>32–36: 14 days	Inferred jointly for transmission trees with $r_{\lambda,low}$ (i.e. trees 1–3 and 7–21) as well as jointly for transmission trees with $r_{\lambda,high}$ (i.e. trees 4–6 and 22–36). SamplingRateChangeTime set according to sampling delay ($t = 0.038$y or $t = 0.019$y).	Inferred jointly for transmission trees with $r_{\lambda,low}$ (i.e. trees 1–3 and 7–21) as well as jointly for transmission trees with $r_{\lambda,high}$ (i.e. trees 4–6 and 22–36).
7	$R_{base,t1} = 3.0$<br>$R_{base,t2} = 2.3$<br>$r_{\lambda,low} = 1.0$<br>$r_{\lambda,high} = 1.666$	1–3: $r_{\lambda,low}$ and $N_{tips} = 50$<br>4–6: $r_{\lambda,high}$ and $N_{tips} = 50$	1–3: none<br>4–6: 14 days	Inferred jointly for transmission trees with $r_{\lambda,low}$ (i.e. trees 1–3) as well as jointly for transmission trees with $r_{\lambda,high}$ (i.e. trees 4–6). SamplingRateChangeTime set for $r_{\lambda,high}$ ($t = 0.038$y).	Inferred jointly for transmission trees with $r_{\lambda,low}$ (i.e. trees 1–3) as well as jointly for transmission trees with $r_{\lambda,high}$ (i.e. trees 4–6).<br>Change in R_{base} inferred by setting a transmission rate change time to $t = 0.058$y.
8	$R_{base,t1} = 1.17$<br>$R_{base,t2} = 0.9$<br>$r_{\lambda,low} = 1.0$<br>$r_{\lambda,high} = 1.6$	1: $r_{\lambda,low}$ and $N_{tips} = 10$<br>2: $r_{\lambda,low}$ and $N_{tips} = 20$<br>3: $r_{\lambda,low}$ and $N_{tips} = 50$<br>4: $r_{\lambda,high}$ and $N_{tips} = 10$<br>5: $r_{\lambda,high}$ and $N_{tips} = 20$<br>6: $r_{\lambda,high}$ and $N_{tips} = 50$	1–3: none<br>4–6: 28 days	Inferred jointly for transmission trees with $r_{\lambda,low}$ (i.e. trees 1–3) as well as jointly for transmission trees with $r_{\lambda,high}$ (i.e. trees 4–6). SamplingRateChangeTime set for $r_{\lambda,high}$ ($t = 0.077$y).	Inferred jointly for transmission trees with $r_{\lambda,low}$ (i.e. trees 1–3) as well as jointly for transmission trees with $r_{\lambda,high}$ (i.e. trees 4–6).<br>Change in R_{base} inferred by setting a transmission rate change time to $t = 0.15$y.
9	$R_{base} = 3.0$<br>$r_{\lambda,low} = 0.8$– 1.2<br>$r_{\lambda,high} = 1.4$– 1.8	1: $r_{\lambda,low} = 0.8$ and $N_{tips} = 25$<br>2: $r_{\lambda,low} = 0.9$ and $N_{tips} = 25$<br>3: $r_{\lambda,low} = 1.0$ and $N_{tips} = 25$<br>4: $r_{\lambda,low} = 1.1$ and $N_{tips} = 25$<br>5: $r_{\lambda,low} = 1.2$ and $N_{tips} = 25$<br>6: $r_{\lambda,high} = 1.4$ and $N_{tips} = 25$<br>7: $r_{\lambda,high} = 1.5$ and $N_{tips} = 25$<br>8: $r_{\lambda,high} = 1.6$ and $N_{tips} = 25$<br>9: $r_{\lambda,high} = 1.7$ and $N_{tips} = 25$<br>10: $r_{\lambda,high} = 1.8$ and $N_{tips} = 25$	1–10: none	Inferred independently for each transmission tree.	Inferred independently for each transmission tree.
10	$R_{base} = 3.0$<br>$r_{\lambda,low} = 0.8$– 1.2<br>$r_{\lambda,high} = 1.4$– 1.8	1: $r_{\lambda,low} = 0.8$ and $N_{tips} = 25$<br>2: $r_{\lambda,low} = 0.9$ and $N_{tips} = 25$<br>3: $r_{\lambda,low} = 1.0$ and $N_{tips} = 25$<br>4: $r_{\lambda,low} = 1.1$ and $N_{tips} = 25$<br>5: $r_{\lambda,low} = 1.2$ and $N_{tips} = 25$<br>6: $r_{\lambda,high} = 1.4$ and $N_{tips} = 25$<br>7: $r_{\lambda,high} = 1.5$ and $N_{tips} = 25$<br>8: $r_{\lambda,high} = 1.6$ and $N_{tips} = 25$<br>9: $r_{\lambda,high} = 1.7$ and $N_{tips} = 25$<br>10: $r_{\lambda,high} = 1.8$ and $N_{tips} = 25$	1–10: none	Inferred jointly for transmission trees with $r_{\lambda,low}$ (i.e. trees 1–5) as well as jointly for transmission trees with $r_{\lambda,high}$ (i.e. trees 6–10).	Inferred jointly for transmission trees with $r_{\lambda,low}$ (i.e. trees 1–5) as well as jointly for transmission trees with $r_{\lambda,high}$ (i.e. trees 6–10).

Supplementary table S2. GISAID acknowledgements.

We gratefully acknowledge the following Authors from the Originating laboratories responsible for obtaining the specimens, as well as the Submitting laboratories where the genome data were generated and shared via GISAID, on which this research is based.

All Submitters of data may be contacted directly via www.gisaid.org

Authors are sorted alphabetically.

Accession ID	Originating Laboratory	Submitting Laboratory	Authors
EPI_ISL_8007868, EPI_ISL_8007948	Bundeswehrkrankenhaus Berlin	Bundeswehr Institute of Microbiology	Alexandra Rehn; Enrico Georgi; Malena Bestehorn-Willmann; Markus Antwerpen; Mathias Walter; Mike Pillukat; Roman Wölfel; Sabine Zange
EPI_ISL_7019047	Charité Universitätsmedizin Berlin, Institute of Virology	Charité Universitätsmedizin Berlin, Institute of Virology	Barbara Mühlemann; Christian Drosten; Julia Schneider; Julia Tesch; Jörn Beheim-Schwarzbach; Talitha Veith; Terry Jones; Tobias Bleicker; Victor M Corman
EPI_ISL_8668212, EPI_ISL_8668213, EPI_ISL_8668214, EPI_ISL_8668215, EPI_ISL_8668217, EPI_ISL_8668218, EPI_ISL_8668219, EPI_ISL_9106582, EPI_ISL_9106583, EPI_ISL_9106584, EPI_ISL_9106585, EPI_ISL_9106586, EPI_ISL_9106587, EPI_ISL_9106588, EPI_ISL_9106589, EPI_ISL_9106590, EPI_ISL_9106591, EPI_ISL_9106592, EPI_ISL_9106593, EPI_ISL_9106594, EPI_ISL_9106595, EPI_ISL_9106596, EPI_ISL_9106597			
see above	Corona-Testzentrum ifp Institut für Produktqualität GmbH	Robert Koch Institute	
EPI_ISL_7760938, EPI_ISL_7760972, EPI_ISL_7760978, EPI_ISL_7760980, EPI_ISL_7760999, EPI_ISL_7761024, EPI_ISL_7761077, EPI_ISL_8223559, EPI_ISL_8223563, EPI_ISL_8223567, EPI_ISL_8223573, EPI_ISL_8223574, EPI_ISL_8223579, EPI_ISL_8223580, EPI_ISL_8223585, EPI_ISL_8223627, EPI_ISL_8223652, EPI_ISL_8223657, EPI_ISL_8223676, EPI_ISL_8223684, EPI_ISL_8223712, EPI_ISL_8223722, EPI_ISL_8223731, EPI_ISL_8223732, EPI_ISL_8223738, EPI_ISL_8223740, EPI_ISL_8223742, EPI_ISL_8223743, EPI_ISL_8223747, EPI_ISL_8223750, EPI_ISL_8413548, EPI_ISL_8413556, EPI_ISL_8413568, EPI_ISL_8413571, EPI_ISL_8413574, EPI_ISL_8413581, EPI_ISL_8413584, EPI_ISL_8413586, EPI_ISL_8413588, EPI_ISL_8664436, EPI_ISL_8664438, EPI_ISL_8664443, EPI_ISL_8664446, EPI_ISL_8664449, EPI_ISL_8664460, EPI_ISL_8664499, EPI_ISL_8664516, EPI_ISL_8664570, EPI_ISL_8664589, EPI_ISL_8664592, EPI_ISL_8664600, EPI_ISL_8664606, EPI_ISL_8664609, EPI_ISL_8664653, EPI_ISL_8664658, EPI_ISL_8664661, EPI_ISL_8664669, EPI_ISL_8664670, EPI_ISL_8664683, EPI_ISL_8664685, EPI_ISL_8681945, EPI_ISL_8681947, EPI_ISL_8681958, EPI_ISL_8682005, EPI_ISL_8682007, EPI_ISL_8682008, EPI_ISL_8682014, EPI_ISL_8682016, EPI_ISL_8682017, EPI_ISL_8682024, EPI_ISL_8682025, EPI_ISL_8682036, EPI_ISL_8682039, EPI_ISL_8682041, EPI_ISL_8682042, EPI_ISL_8682044, EPI_ISL_8682046, EPI_ISL_8682048, EPI_ISL_8682052, EPI_ISL_9110193, EPI_ISL_9110218, EPI_ISL_9110224, EPI_ISL_9110289			
see above	IMD - Institut für Medizinische Diagnostik Berlin-Potsdam	Robert Koch Institute	
EPI_ISL_7750409, EPI_ISL_7750519, EPI_ISL_7750593, EPI_ISL_7768639, EPI_ISL_8222463, EPI_ISL_8222482, EPI_ISL_8222497, EPI_ISL_8222525, EPI_ISL_8222641, EPI_ISL_8222749, EPI_ISL_8222751, EPI_ISL_8222762, EPI_ISL_8222768, EPI_ISL_8222771, EPI_ISL_8222772, EPI_ISL_8222779, EPI_ISL_8222784, EPI_ISL_8222791, EPI_ISL_8222793, EPI_ISL_8222808, EPI_ISL_8222810, EPI_ISL_8222811, EPI_ISL_8222812, EPI_ISL_8222818, EPI_ISL_8222819, EPI_ISL_8222820, EPI_ISL_8222841, EPI_ISL_8222843, EPI_ISL_8222851, EPI_ISL_8222856, EPI_ISL_8222863, EPI_ISL_8222866, EPI_ISL_8222867, EPI_ISL_8222871, EPI_ISL_8222877, EPI_ISL_8222879, EPI_ISL_8222880, EPI_ISL_8222885, EPI_ISL_8222893, EPI_ISL_8222895, EPI_ISL_8222907, EPI_ISL_8222918, EPI_ISL_8222926, EPI_ISL_8222935, EPI_ISL_8222945, EPI_ISL_8222962, EPI_ISL_8222963, EPI_ISL_8222968, EPI_ISL_8222982, EPI_ISL_8222989, EPI_ISL_8223002, EPI_ISL_8223004, EPI_ISL_8223008, EPI_ISL_8223015, EPI_ISL_8223017, EPI_ISL_8223059, EPI_ISL_8223061, EPI_ISL_8223074, EPI_ISL_8228599, EPI_ISL_8228604, EPI_ISL_8228606, EPI_ISL_8228609, EPI_ISL_8228620, EPI_ISL_8228621, EPI_ISL_8228629, EPI_ISL_8228631, EPI_ISL_8228634, EPI_ISL_8228644, EPI_ISL_8228646, EPI_ISL_8228648, EPI_ISL_8228655, EPI_ISL_8228658, EPI_ISL_8228660, EPI_ISL_8228667, EPI_ISL_8228669, EPI_ISL_8228671, EPI_ISL_8228674, EPI_ISL_8228678, EPI_ISL_8228689, EPI_ISL_8228693, EPI_ISL_8228694, EPI_ISL_8228699, EPI_ISL_8228701, EPI_ISL_8228703, EPI_ISL_8228709, EPI_ISL_8228712, EPI_ISL_8228730, EPI_ISL_8228737, EPI_ISL_8228739, EPI_ISL_8228745, EPI_ISL_8228753, EPI_ISL_8228764, EPI_ISL_8228771, EPI_ISL_8228776, EPI_ISL_8228778, EPI_ISL_8228784, EPI_ISL_8228786, EPI_ISL_8228791, EPI_ISL_8228797, EPI_ISL_8228798, EPI_ISL_8228826, EPI_ISL_8228834, EPI_ISL_8228838, EPI_ISL_8228839, EPI_ISL_8228862, EPI_ISL_8228878, EPI_ISL_8228881, EPI_ISL_8228895, EPI_ISL_8228900, EPI_ISL_8228901, EPI_ISL_8228904, EPI_ISL_8228908, EPI_ISL_8228909, EPI_ISL_8228916, EPI_ISL_8228917, EPI_ISL_8228918, EPI_ISL_8228920, EPI_ISL_8228922, EPI_ISL_8669971, EPI_ISL_8669983, EPI_ISL_8669999, EPI_ISL_8670025, EPI_ISL_8670027, EPI_ISL_8670029, EPI_ISL_8670056, EPI_ISL_8670063, EPI_ISL_8670065, EPI_ISL_8670074, EPI_ISL_8670075, EPI_ISL_8670077, EPI_ISL_8670088, EPI_ISL_8670094, EPI_ISL_8670099, EPI_ISL_8670105, EPI_ISL_8670107, EPI_ISL_8670128, EPI_ISL_8670133, EPI_ISL_8670155, EPI_ISL_8670159, EPI_ISL_8670161, EPI_ISL_8670171, EPI_ISL_8670176, EPI_ISL_8670198, EPI_ISL_8670208, EPI_ISL_8670217, EPI_ISL_8670219, EPI_ISL_8670223, EPI_ISL_8670226, EPI_ISL_8670228, EPI_ISL_8670232, EPI_ISL_8670234, EPI_ISL_8670254, EPI_ISL_8670256, EPI_ISL_8670258, EPI_ISL_8670268, EPI_ISL_8670277, EPI_ISL_8670290, EPI_ISL_8670297, EPI_ISL_8670301, EPI_ISL_8670333, EPI_ISL_8670339, EPI_ISL_8670343, EPI_ISL_8670351, EPI_ISL_8670359, EPI_ISL_8670363, EPI_ISL_8670374, EPI_ISL_8670382, EPI_ISL_8670384, EPI_ISL_8670386, EPI_ISL_8670406, EPI_ISL_8670415, EPI_ISL_8670430, EPI_ISL_8670457, EPI_ISL_8670481, EPI_ISL_8670525, EPI_ISL_8670548, EPI_ISL_8670555, EPI_ISL_8670560, EPI_ISL_8670577, EPI_ISL_8670584, EPI_ISL_8670605, EPI_ISL_8670634, EPI_ISL_8670640, EPI_ISL_8670642, EPI_ISL_8670645, EPI_ISL_8670651, EPI_ISL_8670653, EPI_ISL_8670675, EPI_ISL_8670677, EPI_ISL_8670686, EPI_ISL_8670709, EPI_ISL_8670724, EPI_ISL_8670731, EPI_ISL_8670735, EPI_ISL_8670737, EPI_ISL_8670742, EPI_ISL_8670748, EPI_ISL_9055570, EPI_ISL_9055577, EPI_ISL_9055587, EPI_ISL_9055589, EPI_ISL_9055602, EPI_ISL_9055610, EPI_ISL_9055612, EPI_ISL_9055620, EPI_ISL_9055636, EPI_ISL_9055651, EPI_ISL_9055655, EPI_ISL_9055668, EPI_ISL_9055676, EPI_ISL_9055681, EPI_ISL_9055701, EPI_ISL_9055712, EPI_ISL_9055735, EPI_ISL_9055773, EPI_ISL_9055786, EPI_ISL_9055802, EPI_ISL_9055815, EPI_ISL_9055820, EPI_ISL_9055823, EPI_ISL_9105233, EPI_ISL_9105238, EPI_ISL_9105241, EPI_ISL_9105246, EPI_ISL_9105247, EPI_ISL_9105248, EPI_ISL_9105255, EPI_ISL_9105268, EPI_ISL_9105269, EPI_ISL_9105276, EPI_ISL_9105278, EPI_ISL_9105285, EPI_ISL_9105286, EPI_ISL_9105287, EPI_ISL_9105301, EPI_ISL_9105303, EPI_ISL_9105307, EPI_ISL_9105315, EPI_ISL_9105317, EPI_ISL_9105329, EPI_ISL_9105336, EPI_ISL_9105338, EPI_ISL_9105339, EPI_ISL_9105340, EPI_ISL_9105344, EPI_ISL_9105346, EPI_ISL_9105362, EPI_ISL_9105365, EPI_ISL_9105369, EPI_ISL_9105373, EPI_ISL_9105385, EPI_ISL_9105394, EPI_ISL_9105396, EPI_ISL_9105397, EPI_ISL_9105403, EPI_ISL_9105409, EPI_ISL_9105432, EPI_ISL_9105438, EPI_ISL_9105442, EPI_ISL_9105448, EPI_ISL_9105454, EPI_ISL_9105457, EPI_ISL_9105463, EPI_ISL_9105469, EPI_ISL_9105492, EPI_ISL_9105496, EPI_ISL_9105513, EPI_ISL_9105527, EPI_ISL_9105532, EPI_ISL_9105538, EPI_ISL_9105544, EPI_ISL_9105551, EPI_ISL_9105556, EPI_ISL_9105559, EPI_ISL_9212665, EPI_ISL_9212687, EPI_ISL_9212723, EPI_ISL_9212725, EPI_ISL_9212755, EPI_ISL_9212765, EPI_ISL_9212788, EPI_ISL_9212820, EPI_ISL_9212823, EPI_ISL_9212824, EPI_ISL_9212830, EPI_ISL_9212833, EPI_ISL_9212834, EPI_ISL_9212839, EPI_ISL_9212846, EPI_ISL_9212861, EPI_ISL_9212907, EPI_ISL_9212909, EPI_ISL_9212918, EPI_ISL_9212925, EPI_ISL_9212931, EPI_ISL_9212943, EPI_ISL_9212947, EPI_ISL_9212980, EPI_ISL_9213328, EPI_ISL_9213333, EPI_ISL_9213342, EPI_ISL_9213345, EPI_ISL_9213372, EPI_ISL_9213375, EPI_ISL_9213385, EPI_ISL_9213394, EPI_ISL_9213425, EPI_ISL_9213428, EPI_ISL_9213435, EPI_ISL_9213444, EPI_ISL_9213454, EPI_ISL_9213458, EPI_ISL_9213464, EPI_ISL_9213507, EPI_ISL_9213529, EPI_ISL_9213530, EPI_ISL_9213532			
see above	Labor 28 MVZ GmbH	Robert Koch Institute	
EPI_ISL_8503100, EPI_ISL_8503101, EPI_ISL_8503102	Labor Berlin Charite Vivantes GmbH / Institut für Virologie	Charité Universitätsmedizin Berlin, Institut für Virologie	Barbara Mühlemann; Christian Drosten; Julia Schneider; Julia Tesch; Jörn Beheim-Schwarzbach; Talitha Veith; Terry Jones; Tobias Bleicker; Victor M Corman
EPI_ISL_7570571, EPI_ISL_7570572, EPI_ISL_7570573, EPI_ISL_7570587, EPI_ISL_7570588, EPI_ISL_7570589, EPI_ISL_7570597, EPI_ISL_7570598, EPI_ISL_7570599, EPI_ISL_7570603, EPI_ISL_7570606, EPI_ISL_7570607, EPI_ISL_7570608, EPI_ISL_7570609, EPI_ISL_7570610, EPI_ISL_7570613, EPI_ISL_7570618, EPI_ISL_7570619, EPI_ISL_7570621, EPI_ISL_7570622, EPI_ISL_7570623, EPI_ISL_7570624, EPI_ISL_7570626, EPI_ISL_7570630, EPI_ISL_7570631, EPI_ISL_7570632, EPI_ISL_7880690, EPI_ISL_7927210, EPI_ISL_7927232, EPI_ISL_7927243, EPI_ISL_7927253, EPI_ISL_7927264, EPI_ISL_7927275, EPI_ISL_7927401, EPI_ISL_7927409, EPI_ISL_7927418, EPI_ISL_7927433, EPI_ISL_7927434, EPI_ISL_7927435, EPI_ISL_7927436, EPI_ISL_7927439, EPI_ISL_7927477, EPI_ISL_7927490, EPI_ISL_7927503, EPI_ISL_7927532, EPI_ISL_7927544, EPI_ISL_7927555, EPI_ISL_7927570, EPI_ISL_7927580, EPI_ISL_7927582, EPI_ISL_7927592, EPI_ISL_7927605, EPI_ISL_7927621, EPI_ISL_7927622, EPI_ISL_7927623, EPI_ISL_7927624, EPI_ISL_7927647, EPI_ISL_7927657, EPI_ISL_7927671, EPI_ISL_7927677, EPI_ISL_7927749, EPI_ISL_7927757, EPI_ISL_7927767, EPI_ISL_7927779, EPI_ISL_7927788, EPI_ISL_7927797, EPI_ISL_7927801, EPI_ISL_7927811, EPI_ISL_7927816, EPI_ISL_7927826, EPI_ISL_7927866, EPI_ISL_7927876, EPI_ISL_7927886, EPI_ISL_7927906, EPI_ISL_7927916, EPI_ISL_7927923, EPI_ISL_7927933, EPI_ISL_7927943, EPI_ISL_7927953, EPI_ISL_7927961, EPI_ISL_7927971, EPI_ISL_7927981, EPI_ISL_7928013, EPI_ISL_7928023, EPI_ISL_7928045, EPI_ISL_7928054, EPI_ISL_7928065, EPI_ISL_7928076, EPI_ISL_8099070, EPI_ISL_8099086, EPI_ISL_8099088, EPI_ISL_8099092, EPI_ISL_8099099, EPI_ISL_8099102, EPI_ISL_8099107, EPI_ISL_8099109, EPI_ISL_8099113, EPI_ISL_8099115, EPI_ISL_8099125, EPI_ISL_8099127, EPI_ISL_8099132, EPI_ISL_8099136, EPI_ISL_8099141, EPI_ISL_8099146, EPI_ISL_8099148, EPI_ISL_8099150, EPI_ISL_8099152, EPI_ISL_8099154, EPI_ISL_8099156, EPI_ISL_8099158, EPI_ISL_8099162, EPI_ISL_8099166, EPI_ISL_8099170, EPI_ISL_8099172, EPI_ISL_8099176, EPI_ISL_8099177, EPI_ISL_8099184, EPI_ISL_8099188, EPI_ISL_8099189, EPI_ISL_8099191, EPI_ISL_8099194, EPI_ISL_8099196, EPI_ISL_8099198, EPI_ISL_8099200, EPI_ISL_8099202, EPI_ISL_8099204, EPI_ISL_8099206, EPI_ISL_8099208, EPI_ISL_8099210, EPI_ISL_8099214, EPI_ISL_8099221, EPI_ISL_8099222, EPI_ISL_8099226, EPI_ISL_8099229, EPI_ISL_8315016, EPI_ISL_8315019, EPI_ISL_8315023, EPI_ISL_8315024, EPI_ISL_8315025, EPI_ISL_8315026, EPI_ISL_8315027, EPI_ISL_8315028, EPI_ISL_8315029, EPI_ISL_8315030, EPI_ISL_8315032, EPI_ISL_8315033, EPI_ISL_8315035, EPI_ISL_8315036, EPI_ISL_8315039, EPI_ISL_8315040, EPI_ISL_8315042, EPI_ISL_8315043, EPI_ISL_8315044, EPI_ISL_8315046, EPI_ISL_8315047, EPI_ISL_8315048, EPI_ISL_8315049, EPI_ISL_8315055, EPI_ISL_8315056, EPI_ISL_8315057, EPI_ISL_8315058, EPI_ISL_8315060, EPI_ISL_8315061, EPI_ISL_8315062, EPI_ISL_8315063, EPI_ISL_8927050, EPI_ISL_8927052, EPI_ISL_8927055, EPI_ISL_8927056, EPI_ISL_8927059, EPI_ISL_8927065, EPI_ISL_8927066, EPI_ISL_8927067, EPI_ISL_8927073, EPI_ISL_8927083, EPI_ISL_8927099, EPI_ISL_8927108, EPI_ISL_8927115, EPI_ISL_8927120, EPI_ISL_8927126, EPI_ISL_8927328, EPI_ISL_8927338, EPI_ISL_8927340, EPI_ISL_8927341, EPI_ISL_8927345, EPI_ISL_8927346, EPI_ISL_8927347, EPI_ISL_8927359, EPI_ISL_8927371, EPI_ISL_8927377, EPI_ISL_8927388, EPI_ISL_8927425, EPI_ISL_8927430, EPI_ISL_8927444, EPI_ISL_8927450, EPI_ISL_8927457, EPI_ISL_8927482, EPI_ISL_8927495, EPI_ISL_8927503, EPI_ISL_8927509			
see above	Labor Berlin Charité Vivantes GmbH / Institut für Virologie	Charité Universitätsmedizin Berlin, Institut für Virologie/Labor Berlin	Barbara Mühlemann; Christian Drosten; Christine Stephan; Peter Menzel; Rolf Schwarzer; Terry Jones; Victor M Corman
EPI_ISL_7429121, EPI_ISL_7429150, EPI_ISL_7437704, EPI_ISL_7437711, EPI_ISL_7437736, EPI_ISL_7437767, EPI_ISL_7437774, EPI_ISL_7437916, EPI_ISL_7437944, EPI_ISL_7437950, EPI_ISL_7437957, EPI_ISL_7437965, EPI_ISL_7437969, EPI_ISL_7437974, EPI_ISL_7437977, EPI_ISL_7437995, EPI_ISL_7438753, EPI_ISL_7438756, EPI_ISL_7438770, EPI_ISL_7438808, EPI_ISL_7438809, EPI_ISL_7438816, EPI_ISL_7438840, EPI_ISL_7749653, EPI_ISL_7749666, EPI_ISL_7749675, EPI_ISL_7749693, EPI_ISL_7749694, EPI_ISL_7749697, EPI_ISL_7749701, EPI_ISL_7749706, EPI_ISL_7749709, EPI_ISL_7749710, EPI_ISL_7749712, EPI_ISL_7749713, EPI_ISL_7749715, EPI_ISL_7750071, EPI_ISL_7750074, EPI_ISL_7750715, EPI_ISL_7750719, EPI_ISL_7750723, EPI_ISL_7750733, EPI_ISL_7750735, EPI_ISL_7750747, EPI_ISL_7750749, EPI_ISL_7750752, EPI_ISL_7750753, EPI_ISL_7750754, EPI_ISL_7750755, EPI_ISL_7750756, EPI_ISL_7750757, EPI_ISL_7750763, EPI_ISL_7750765, EPI_ISL_7750767, EPI_ISL_7750768, EPI_ISL_7750770, EPI_ISL_7750774, EPI_ISL_7750776, EPI_ISL_7750778, EPI_ISL_7750779, EPI_ISL_7750803, EPI_ISL_7750815, EPI_ISL_7750817, EPI_ISL_7750820, EPI_ISL_7750827, EPI_ISL_7751414, EPI_ISL_7751424, EPI_ISL_7751431, EPI_ISL_7751440, EPI_ISL_7751446, EPI_ISL_7751447, EPI_ISL_7751457, EPI_ISL_7751462, EPI_ISL_7751516, EPI_ISL_7751517, EPI_ISL_7751520, EPI_ISL_7751523, EPI_ISL_7751529, EPI_ISL_7751532, EPI_ISL_7751536, EPI_ISL_7751538, EPI_ISL_7751545, EPI_ISL_7751549, EPI_ISL_7751553, EPI_ISL_7751555, EPI_ISL_7751558, EPI_ISL_7751561, EPI_ISL_7751564, EPI_ISL_7751566, EPI_ISL_7751567, EPI_ISL_7751570, EPI_ISL_7751572, EPI_ISL_7751574, EPI_ISL_7751575, EPI_ISL_7751578, EPI_ISL_7751581, EPI_ISL_7751583, EPI_ISL_7751823, EPI_ISL_7751836, EPI_ISL_7751841, EPI_ISL_7751849, EPI_ISL_7751883, EPI_ISL_7752811, EPI_ISL_7752818, EPI_ISL_7752819, EPI_ISL_7752821, EPI_ISL_7752822, EPI_ISL_7752824, EPI_ISL_7752825, EPI_ISL_7752828, EPI_ISL_7752830, EPI_ISL_7752831, EPI_ISL_7752833, EPI_ISL_7752834, EPI_ISL_7752836, EPI_ISL_7752837, EPI_ISL_7752840, EPI_ISL_7752841, EPI_ISL_7752845, EPI_ISL_7752847, EPI_ISL_7752848, EPI_ISL_7752850, EPI_ISL_7752851, EPI_ISL_7752854, EPI_ISL_7752856, EPI_ISL_7752857, EPI_ISL_7752860, EPI_ISL_7752863, EPI_ISL_7752866, EPI_ISL_7752867, EPI_ISL_7752869, EPI_ISL_7752871, EPI_ISL_7752876, EPI_ISL_7752878, EPI_ISL_7752881, EPI_ISL_7752882, EPI_ISL_7752885, EPI_ISL_7752887, EPI_ISL_7753342, EPI_ISL_7754336, EPI_ISL_7754337, EPI_ISL_7754340, EPI_ISL_7754342, EPI_ISL_7754343, EPI_ISL_7754344, EPI_ISL_7754345, EPI_ISL_7754346, EPI_ISL_7754347, EPI_ISL_7754348, EPI_ISL_7754349, EPI_ISL_7754350, EPI_ISL_7754351, EPI_ISL_7754353, EPI_ISL_7754354, EPI_ISL_7754356, EPI_ISL_7754357, EPI_ISL_7754365, EPI_ISL_7754366, EPI_ISL_7754367, EPI_ISL_7754368, EPI_ISL_7754369, EPI_ISL_7754370, EPI_ISL_7754373, EPI_ISL_7754374, EPI_ISL_7754375, EPI_ISL_7754376, EPI_ISL_7754378, EPI_ISL_7754382, EPI_ISL_7754383, EPI_ISL_7754384, EPI_ISL_7754385, EPI_ISL_7754387, EPI_ISL_7754389, EPI_ISL_7754392, EPI_ISL_7754393, EPI_ISL_7754395, EPI_ISL_7754396, EPI_ISL_7754397, EPI_ISL_7754399, EPI_ISL_7754400, EPI_ISL_7754401, EPI_ISL_7754402, EPI_ISL_7754404, EPI_ISL_7754405, EPI_ISL_7754410, EPI_ISL_7754411, EPI_ISL_7754415, EPI_ISL_7754416, EPI_ISL_7754417, EPI_ISL_7754418, EPI_ISL_7754419, EPI_ISL_7754420, EPI_ISL_7755062, EPI_ISL_7755064, EPI_ISL_7755067, EPI_ISL_7755068, EPI_ISL_7755074, EPI_ISL_7755094, EPI_ISL_7755095, EPI_ISL_7755096, EPI_ISL_7755101, EPI_ISL_7755450, EPI_ISL_7755451, EPI_ISL_7755452, EPI_ISL_7755453, EPI_ISL_7755454, EPI_ISL_7755455, EPI_ISL_7755456, EPI_ISL_7755458, EPI_ISL_7755459, EPI_ISL_7755460, EPI_ISL_7755461, EPI_ISL_7755462, EPI_ISL_7755463, EPI_ISL_7755464, EPI_ISL_7755465, EPI_ISL_7755466, EPI_ISL_7755467, EPI_ISL_7755469, EPI_ISL_7755470, EPI_ISL_7755471, EPI_ISL_7755472, EPI_ISL_7755473, EPI_ISL_7755474, EPI_ISL_7755475, EPI_ISL_7755476, EPI_ISL_7755477, EPI_ISL_7755478, EPI_ISL_7755480, EPI_ISL_7755481, EPI_ISL_7755482, EPI_ISL_7755483, EPI_ISL_7755484, EPI_ISL_7755485, EPI_ISL_7755486, EPI_ISL_7755488, EPI_ISL_7755489, EPI_ISL_7755490, EPI_ISL_7755491, EPI_ISL_7755492, EPI_ISL_7755493, EPI_ISL_7755494, EPI_ISL_7755495, EPI_ISL_7755496, EPI_ISL_7755497, EPI_ISL_7755498, EPI_ISL_7755499, EPI_ISL_7755500, EPI_ISL_7755502, EPI_ISL_7755504, EPI_ISL_7755818, EPI_ISL_7755820, EPI_ISL_7755821, EPI_ISL_7755822, EPI_ISL_7755823, EPI_ISL_7755824, EPI_ISL_7755825, EPI_ISL_7755827, EPI_ISL_7755828, EPI_ISL_7755829, EPI_ISL_7755830, EPI_ISL_7755831, EPI_ISL_7755832, EPI_ISL_7755833, EPI_ISL_7755834, EPI_ISL_7755835, EPI_ISL_7755836, EPI_ISL_7755837, EPI_ISL_7755838, EPI_ISL_7755839, EPI_ISL_7755840, EPI_ISL_7755841, EPI_ISL_7755842, EPI_ISL_7755843, EPI_ISL_7755844, EPI_ISL_7755845, EPI_ISL_7755846, EPI_ISL_7755881, EPI_ISL_7755882, EPI_ISL_7755883, EPI_ISL_7755885, EPI_ISL_7755886, EPI_ISL_7755887, EPI_ISL_7755888, EPI_ISL_7755889, EPI_ISL_7755891, EPI_ISL_7755892, EPI_ISL_7755893, EPI_ISL_7755894, EPI_ISL_7755896, EPI_ISL_7755897, EPI_ISL_7755898, EPI_ISL_7755899, EPI_ISL_7755900, EPI_ISL_7755901, EPI_ISL_7755902, EPI_ISL_7755903, EPI_ISL_7755905, EPI_ISL_7755908, EPI_ISL_7755909, EPI_ISL_7755910, EPI_ISL_7755945, EPI_ISL_7755946, EPI_ISL_7755954, EPI_ISL_7755955, EPI_ISL_7755958, EPI_ISL_7755959, EPI_ISL_7755960, EPI_ISL_7755962, EPI_ISL_7755964, EPI_ISL_7755967, EPI_ISL_7755968, EPI_ISL_7755970, EPI_ISL_7756050, EPI_ISL_7756052, EPI_ISL_7758082, EPI_ISL_7763038, EPI_ISL_7763045, EPI_ISL_7763088, EPI_ISL_7763095, EPI_ISL_7763106, EPI_ISL_7763112, EPI_ISL_7763123, EPI_ISL_7763128, EPI_ISL_7763131, EPI_ISL_7763134, EPI_ISL_7763136, EPI_ISL_7763139, EPI_ISL_7763141, EPI_ISL_7763144, EPI_ISL_7763147, EPI_ISL_7763149, EPI_ISL_7763152, EPI_ISL_7763154, EPI_ISL_7763157, EPI_ISL_7763160, EPI_ISL_7763163, EPI_ISL_7763168, EPI_ISL_7763171, EPI_ISL_7763173, EPI_ISL_7763175, EPI_ISL_7763178, EPI_ISL_7763184, EPI_ISL_7763186, EPI_ISL_7763262, EPI_ISL_7763265, EPI_ISL_7763269, EPI_ISL_7763271, EPI_ISL_7763273, EPI_ISL_7763275, EPI_ISL_7763277, EPI_ISL_7763279, EPI_ISL_7763285, EPI_ISL_7763287, EPI_ISL_7763290, EPI_ISL_7763293, EPI_ISL_7763296, EPI_ISL_7763298, EPI_ISL_7763301, EPI_ISL_7763304, EPI_ISL_7763306, EPI_ISL_7763308, EPI_ISL_7763315, EPI_ISL_7763318, EPI_ISL_7763320, EPI_ISL_7763322, EPI_ISL_7763328, EPI_ISL_7763331, EPI_ISL_7763412, EPI_ISL_7763415, EPI_ISL_7763419, EPI_ISL_7763421, EPI_ISL_7763424, EPI_ISL_7763427, EPI_ISL_7763429, EPI_ISL_7763432, EPI_ISL_7763436, EPI_ISL_7763438, EPI_ISL_7763441, EPI_ISL_7763444, EPI_ISL_7763447, EPI_ISL_7763450, EPI_ISL_7763453, EPI_ISL_7763459, EPI_ISL_7763462, EPI_ISL_7763464, EPI_ISL_7763467, EPI_ISL_7763470, EPI_ISL_7763473, EPI_ISL_7763476, EPI_ISL_7763479, EPI_ISL_7763481, EPI_ISL_7763484, EPI_ISL_7763487, EPI_ISL_7763490, EPI_ISL_7763493, EPI_ISL_7763496, EPI_ISL_7763499, EPI_ISL_7763502, EPI_ISL_7763504, EPI_ISL_7763507, EPI_ISL_7763511, EPI_ISL_7763512, EPI_ISL_7763515, EPI_ISL_7763516, EPI_ISL_7763517, EPI_ISL_7763518, EPI_ISL_7763519, EPI_ISL_7763521, EPI_ISL_7763522, EPI_ISL_7763523, EPI_ISL_7763524, EPI_ISL_7763525, EPI_ISL_7763526, EPI_ISL_7763528, EPI_ISL_7763529, EPI_ISL_7763530, EPI_ISL_7763531, EPI_ISL_7763532, EPI_ISL_7763533, EPI_ISL_7763535, EPI_ISL_7763536, EPI_ISL_7763537, EPI_ISL_7763538, EPI_ISL_7763539, EPI_ISL_7763540, EPI_ISL_7763542, EPI_ISL_7763546, EPI_ISL_7763557, EPI_ISL_7763561, EPI_ISL_7763563, EPI_ISL_7763564, EPI_ISL_7763567, EPI_ISL_7763569, EPI_ISL_7763572, EPI_ISL_7763576, EPI_ISL_7763578, EPI_ISL_7763581, EPI_ISL_7763583, EPI_ISL_7763586, EPI_ISL_7763589, EPI_ISL_7763592, EPI_ISL_7763595, EPI_ISL_7763597, EPI_ISL_7763600, EPI_ISL_7763603, EPI_ISL_7763606, EPI_ISL_7763610, EPI_ISL_7763612, EPI_ISL_7763615, EPI_ISL_7763618, EPI_ISL_7763621, EPI_ISL_7763624, EPI_ISL_7763627, EPI_ISL_7764370, EPI_ISL_7764838, EPI_ISL_7764992, EPI_ISL_7765271, EPI_ISL_7765272, EPI_ISL_7765273, EPI_ISL_7765274, EPI_ISL_7765277, EPI_ISL_7765278, EPI_ISL_7765279, EPI_ISL_7765281, EPI_ISL_7765282, EPI_ISL_7765283, EPI_ISL_7765285, EPI_ISL_7765288, EPI_ISL_7765289, EPI_ISL_7765290, EPI_ISL_7765291, EPI_ISL_7765294, EPI_ISL_7765295, EPI_ISL_7765296, EPI_ISL_7765300, EPI_ISL_7767242, EPI_ISL_7767244, EPI_ISL_7767246, EPI_ISL_7767247, EPI_ISL_7767249, EPI_ISL_7767251, EPI_ISL_7767253, EPI_ISL_7767254, EPI_ISL_7767257, EPI_ISL_7767259, EPI_ISL_7767260, EPI_ISL_7767262, EPI_ISL_7767263, EPI_ISL_7767265, EPI_ISL_7767268, EPI_ISL_7767270, EPI_ISL_7767271, EPI_ISL_7767272, EPI_ISL_7767274, EPI_ISL_7767276, EPI_ISL_7767277, EPI_ISL_7767279, EPI_ISL_7767282, EPI_ISL_7767283, EPI_ISL_7767285, EPI_ISL_7767286, EPI_ISL_7767288, EPI_ISL_7767290, EPI_ISL_7767294, EPI_ISL_7767302, EPI_ISL_7767307, EPI_ISL_7767310, EPI_ISL_7767316, EPI_ISL_7767324, EPI_ISL_7767325, EPI_ISL_7767330, EPI_ISL_7767331, EPI_ISL_7767333, EPI_ISL_7767335, EPI_ISL_7767336, EPI_ISL_7767339, EPI_ISL_7767340, EPI_ISL_7767342, EPI_ISL_7767345, EPI_ISL_7767347, EPI_ISL_7767350, EPI_ISL_7767351, EPI_ISL_7767353, EPI_ISL_7767355, EPI_ISL_7767357, EPI_ISL_7767358, EPI_ISL_7767361, EPI_ISL_7767363, EPI_ISL_7767364, EPI_ISL_7767366, EPI_ISL_7767368, EPI_ISL_7767372, EPI_ISL_7767373, EPI_ISL_7767375, EPI_ISL_7767378, EPI_ISL_7767380, EPI_ISL_7767382, EPI_ISL_7767386, EPI_ISL_7767388, EPI_ISL_7767389, EPI_ISL_7767392, EPI_ISL_7767394, EPI_ISL_7767395, EPI_ISL_7767397, EPI_ISL_7767398, EPI_ISL_7767400, EPI_ISL_7767403, EPI_ISL_7767404, EPI_ISL_7767406, EPI_ISL_7767408, EPI_ISL_7767409, EPI_ISL_7767414, EPI_ISL_7767415, EPI_ISL_7767416, EPI_ISL_7767417, EPI_ISL_7767418, EPI_ISL_7767419, EPI_ISL_7767421, EPI_ISL_7767422, EPI_ISL_7767423, EPI_ISL_7767425, EPI_ISL_7767427, EPI_ISL_7767430, EPI_ISL_7767433, EPI_ISL_7767434, EPI_ISL_7767436, EPI_ISL_7767437, EPI_ISL_7767439, EPI_ISL_7767440, EPI_ISL_7767442, EPI_ISL_7767445, EPI_ISL_7767446, EPI_ISL_7767448, EPI_ISL_7767449, EPI_ISL_7767451, EPI_ISL_7767453, EPI_ISL_7767455, EPI_ISL_7767459, EPI_ISL_7767460, EPI_ISL_7767462, EPI_ISL_7767464, EPI_ISL_7767466, EPI_ISL_7767468, EPI_ISL_7767469, EPI_ISL_7767471, EPI_ISL_7767473, EPI_ISL_7767476, EPI_ISL_7767479, EPI_ISL_7767480, EPI_ISL_7767482, EPI_ISL_7767484, EPI_ISL_7767485, EPI_ISL_7767486, EPI_ISL_7767488, EPI_ISL_7767490, EPI_ISL_7767491, EPI_ISL_7767492, EPI_ISL_7767493, EPI_ISL_7767495, EPI_ISL_7767496, EPI_ISL_7767499, EPI_ISL_7767502, EPI_ISL_7767503, EPI_ISL_7767505, EPI_ISL_7767506, EPI_ISL_7767509, EPI_ISL_7767512, EPI_ISL_7767513, EPI_ISL_7767515, EPI_ISL_7767519, EPI_ISL_7767525, EPI_ISL_7767526, EPI_ISL_7767529, EPI_ISL_7767532, EPI_ISL_7767538, EPI_ISL_7767541, EPI_ISL_7767542, EPI_ISL_7767544, EPI_ISL_7767545, EPI_ISL_7767548, EPI_ISL_7767552, EPI_ISL_7767554, EPI_ISL_7767556, EPI_ISL_7767560, EPI_ISL_7767564, EPI_ISL_7767568, EPI_ISL_7767570, EPI_ISL_7767572, EPI_ISL_7767574, EPI_ISL_7767576, EPI_ISL_7767577, EPI_ISL_7767580, EPI_ISL_7767582, EPI_ISL_7767588, EPI_ISL_7767593, EPI_ISL_7767596, EPI_ISL_7767598, EPI_ISL_7767601, EPI_ISL_7767604, EPI_ISL_7767606, EPI_ISL_7767608, EPI_ISL_7767610, EPI_ISL_7767615, EPI_ISL_7767618, EPI_ISL_7767620, EPI_ISL_7767626, EPI_ISL_7767628, EPI_ISL_7767631, EPI_ISL_7767634, EPI_ISL_7767636, EPI_ISL_7767638, EPI_ISL_7769918, EPI_ISL_7769921, EPI_ISL_7769927, EPI_ISL_7769929, EPI_ISL_7769930, EPI_ISL_7769935, EPI_ISL_7769938, EPI_ISL_7769940, EPI_ISL_7769941, EPI_ISL_7769943, EPI_ISL_7769947, EPI_ISL_7769950, EPI_ISL_7769953, EPI_ISL_7769957, EPI_ISL_7769959, EPI_ISL_7769962, EPI_ISL_7769964, EPI_ISL_7769966, EPI_ISL_7769971, EPI_ISL_7769973, EPI_ISL_7769974, EPI_ISL_7769976, EPI_ISL_7769978, EPI_ISL_7769979, EPI_ISL_7769982, EPI_ISL_7769983, EPI_ISL_7769985, EPI_ISL_7769987, EPI_ISL_7769989, EPI_ISL_7769992, EPI_ISL_7769994, EPI_ISL_7769995, EPI_ISL_7769997, EPI_ISL_7769999, EPI_ISL_7770000, EPI_ISL_7770002, EPI_ISL_7770005, EPI_ISL_7770006, EPI_ISL_7770008, EPI_ISL_7770010, EPI_ISL_7770011, EPI_ISL_7770013, EPI_ISL_7770015, EPI_ISL_7770017, EPI_ISL_7770020, EPI_ISL_7770021, EPI_ISL_7770023, EPI_ISL_7770025, EPI_ISL_7770026, EPI_ISL_7770028, EPI_ISL_7770030, EPI_ISL_7770032, EPI_ISL_7770034, EPI_ISL_7770035, EPI_ISL_7770037, EPI_ISL_7770039, EPI_ISL_7770042, EPI_ISL_7770044, EPI_ISL_7770046, EPI_ISL_7770047, EPI_ISL_7770049, EPI_ISL_7770051, EPI_ISL_7770052, EPI_ISL_7770055, EPI_ISL_7770057, EPI_ISL_7770058, EPI_ISL_7770060, EPI_ISL_7770062, EPI_ISL_7770063, EPI_ISL_7770065, EPI_ISL_7770068, EPI_ISL_7770069, EPI_ISL_7770071, EPI_ISL_7770073, EPI_ISL_7770074, EPI_ISL_7770076, EPI_ISL_7770169, EPI_ISL_7770170, EPI_ISL_7770174, EPI_ISL_7770176, EPI_ISL_7770177, EPI_ISL_7770179, EPI_ISL_7770180, EPI_ISL_7770182, EPI_ISL_7770184, EPI_ISL_7770186, EPI_ISL_7770188, EPI_ISL_7770189, EPI_ISL_7770191, EPI_ISL_7770193, EPI_ISL_7770194, EPI_ISL_7770195, EPI_ISL_7770198, EPI_ISL_7770200, EPI_ISL_7770201, EPI_ISL_7770203, EPI_ISL_7770205, EPI_ISL_7770206, EPI_ISL_7770209, EPI_ISL_7770210, EPI_ISL_7770214, EPI_ISL_7770217, EPI_ISL_7770218, EPI_ISL_7770221, EPI_ISL_7770224, EPI_ISL_7770225, EPI_ISL_7770227, EPI_ISL_7770228, EPI_ISL_7770230, EPI_ISL_7770232, EPI_ISL_7770234, EPI_ISL_7770236, EPI_ISL_7770237, EPI_ISL_7770239, EPI_ISL_7770241, EPI_ISL_7770242, EPI_ISL_7770245, EPI_ISL_7770246, EPI_ISL_7770248, EPI_ISL_7770249, EPI_ISL_7770251, EPI_ISL_7770253, EPI_ISL_7770254, EPI_ISL_7770257, EPI_ISL_7770258, EPI_ISL_7770260, EPI_ISL_7770262, EPI_ISL_7770263, EPI_ISL_7770265, EPI_ISL_7770267, EPI_ISL_7770269, EPI_ISL_7770270, EPI_ISL_7770271, EPI_ISL_7770273, EPI_ISL_7770278, EPI_ISL_7770280, EPI_ISL_7770282, EPI_ISL_7770283, EPI_ISL_7770287, EPI_ISL_7770290, EPI_ISL_7770292, EPI_ISL_7770294, EPI_ISL_7770296, EPI_ISL_7770297, EPI_ISL_7770299, EPI_ISL_7770301, EPI_ISL_7770305, EPI_ISL_7770309, EPI_ISL_7770311, EPI_ISL_7770313, EPI_ISL_7770320, EPI_ISL_7770323, EPI_ISL_7770325, EPI_ISL_7770328, EPI_ISL_7770330, EPI_ISL_7770331, EPI_ISL_7770335, EPI_ISL_8220734, EPI_ISL_8220738, EPI_ISL_8220743, EPI_ISL_8223759, EPI_ISL_8223775, EPI_ISL_8223776, EPI_ISL_8223777, EPI_ISL_8223780, EPI_ISL_8223782, EPI_ISL_8223783, EPI_ISL_8223784, EPI_ISL_8223786, EPI_ISL_8223792, EPI_ISL_8223793, EPI_ISL_8223794, EPI_ISL_8223796, EPI_ISL_8223797, EPI_ISL_8223798, EPI_ISL_8223800, EPI_ISL_8223802, EPI_ISL_8223804, EPI_ISL_8223805, EPI_ISL_8223806, EPI_ISL_8223808, EPI_ISL_8223809, EPI_ISL_8223811, EPI_ISL_8223812, EPI_ISL_8223813, EPI_ISL_8223815, EPI_ISL_8223816, EPI_ISL_8223817, EPI_ISL_8223818, EPI_ISL_8223820, EPI_ISL_8223823, EPI_ISL_8223825, EPI_ISL_8223826, EPI_ISL_8223829, EPI_ISL_8223830, EPI_ISL_8223834, EPI_ISL_8223835, EPI_ISL_8223836, EPI_ISL_8224152, EPI_ISL_8224154, EPI_ISL_8224160, EPI_ISL_8224161, EPI_ISL_8224163, EPI_ISL_8224164, EPI_ISL_8224166, EPI_ISL_8224168, EPI_ISL_8226038, EPI_ISL_8226043, EPI_ISL_8226044, EPI_ISL_8226049, EPI_ISL_8226051, EPI_ISL_8226059, EPI_ISL_8226063, EPI_ISL_8226064, EPI_ISL_8226070, EPI_ISL_8226073, EPI_ISL_8226095, EPI_ISL_8226096, EPI_ISL_8226097, EPI_ISL_8226098, EPI_ISL_8226099, EPI_ISL_8226101, EPI_ISL_8226102, EPI_ISL_8226103, EPI_ISL_8226105, EPI_ISL_8226106, EPI_ISL_8226109, EPI_ISL_8226111, EPI_ISL_8226112, EPI_ISL_8226113, EPI_ISL_8226114, EPI_ISL_8226115, EPI_ISL_8226119, EPI_ISL_8226120, EPI_ISL_8226148, EPI_ISL_8226149, EPI_ISL_8228194, EPI_ISL_8228196, EPI_ISL_8228198, EPI_ISL_8228200, EPI_ISL_8228204, EPI_ISL_8228205, EPI_ISL_8228206, EPI_ISL_8228207, EPI_ISL_8228208, EPI_ISL_8228209, EPI_ISL_8228210, EPI_ISL_8228211, EPI_ISL_8228218, EPI_ISL_8228223, EPI_ISL_8228224, EPI_ISL_8228225, EPI_ISL_8228227, EPI_ISL_8228230, EPI_ISL_8228232, EPI_ISL_8228235, EPI_ISL_8228236, EPI_ISL_8228238, EPI_ISL_8228239, EPI_ISL_8228306, EPI_ISL_8228309, EPI_ISL_8228397, EPI_ISL_8228401, EPI_ISL_8228402, EPI_ISL_8228403, EPI_ISL_8234537, EPI_ISL_8234540, EPI_ISL_8234544, EPI_ISL_8234547, EPI_ISL_8234551, EPI_ISL_8234566, EPI_ISL_8234573, EPI_ISL_8234586, EPI_ISL_8234589, EPI_ISL_8234596, EPI_ISL_8234602, EPI_ISL_8234606, EPI_ISL_8234609, EPI_ISL_8234629, EPI_ISL_8234635, EPI_ISL_8234637, EPI_ISL_8234640, EPI_ISL_8234645, EPI_ISL_8234648, EPI_ISL_8234651, EPI_ISL_8234653, EPI_ISL_8234788, EPI_ISL_8234900, EPI_ISL_8234902, EPI_ISL_8234910, EPI_ISL_8234913, EPI_ISL_8234920, EPI_ISL_8234922, EPI_ISL_8234924, EPI_ISL_8234926, EPI_ISL_8234932, EPI_ISL_8234937, EPI_ISL_8234941, EPI_ISL_8234959, EPI_ISL_8234962, EPI_ISL_8234967, EPI_ISL_8234981, EPI_ISL_8234984, EPI_ISL_8234992, EPI_ISL_8235027, EPI_ISL_8235030, EPI_ISL_8235059, EPI_ISL_8235067, EPI_ISL_8235071, EPI_ISL_8235086, EPI_ISL_8235090, EPI_ISL_8248343, EPI_ISL_8248346, EPI_ISL_8248347, EPI_ISL_8248354, EPI_ISL_8248371, EPI_ISL_8248380, EPI_ISL_8248382, EPI_ISL_8248508, EPI_ISL_8248513, EPI_ISL_8248515, EPI_ISL_8248516, EPI_ISL_8248517, EPI_ISL_8248525, EPI_ISL_8248526, EPI_ISL_8248645, EPI_ISL_8248667, EPI_ISL_8248805, EPI_ISL_8248807, EPI_ISL_8248812, EPI_ISL_8248819, EPI_ISL_8248822, EPI_ISL_8248847, EPI_ISL_8248849, EPI_ISL_8248855, EPI_ISL_8248856, EPI_ISL_8248878, EPI_ISL_8249210, EPI_ISL_8411838, EPI_ISL_8411847, EPI_ISL_8411859, EPI_ISL_8411860, EPI_ISL_8412652, EPI_ISL_8412664, EPI_ISL_8412667, EPI_ISL_8412678, EPI_ISL_8412687, EPI_ISL_8412692, EPI_ISL_8412706, EPI_ISL_8412708, EPI_ISL_8412828, EPI_ISL_8412936, EPI_ISL_8412975, EPI_ISL_8665119, EPI_ISL_8665702, EPI_ISL_8665756, EPI_ISL_8665879, EPI_ISL_8672879, EPI_ISL_8672914, EPI_ISL_8672938, EPI_ISL_8672953, EPI_ISL_8672979, EPI_ISL_8680849, EPI_ISL_8680850, EPI_ISL_8680860, EPI_ISL_8680863, EPI_ISL_8682260, EPI_ISL_9056005, EPI_ISL_9103784, EPI_ISL_9106375, EPI_ISL_9106379, EPI_ISL_9106381, EPI_ISL_9106384, EPI_ISL_9106387, EPI_ISL_9106388, EPI_ISL_9106389, EPI_ISL_9106393			
see above	MDI Limbach Berlin GmbH; MVZ Labor Berlin	Robert Koch Institute	
EPI_ISL_7116918, EPI_ISL_7418264, EPI_ISL_7418265, EPI_ISL_7418266, EPI_ISL_7418267, EPI_ISL_7418268, EPI_ISL_7418269, EPI_ISL_7418270, EPI_ISL_7418271, EPI_ISL_7418272, EPI_ISL_7418273, EPI_ISL_7418274, EPI_ISL_7418275, EPI_ISL_7418276, EPI_ISL_7418277, EPI_ISL_7418278, EPI_ISL_7418279, EPI_ISL_7418280, EPI_ISL_7418281, EPI_ISL_7418282, EPI_ISL_7418283, EPI_ISL_7418284, EPI_ISL_7418285, EPI_ISL_7418286, EPI_ISL_7418287, EPI_ISL_7418288, EPI_ISL_7418289, EPI_ISL_7418290, EPI_ISL_7418291, EPI_ISL_7418292, EPI_ISL_7418293, EPI_ISL_7418294, EPI_ISL_7418295, EPI_ISL_7418296, EPI_ISL_7418297, EPI_ISL_7418298, EPI_ISL_7418299, EPI_ISL_7418301, EPI_ISL_7418302, EPI_ISL_7418303, EPI_ISL_7432665, EPI_ISL_7432666, EPI_ISL_7432675, EPI_ISL_7432680, EPI_ISL_7432681, EPI_ISL_7750737, EPI_ISL_7755111, EPI_ISL_7755112, EPI_ISL_7755113, EPI_ISL_7755114, EPI_ISL_7755115, EPI_ISL_7755116, EPI_ISL_7756752, EPI_ISL_7756753, EPI_ISL_7756754, EPI_ISL_7756755, EPI_ISL_7756756, EPI_ISL_7756757, EPI_ISL_7756758, EPI_ISL_7756759, EPI_ISL_7756760, EPI_ISL_7756761, EPI_ISL_8245915, EPI_ISL_8245917, EPI_ISL_8245920, EPI_ISL_8245922, EPI_ISL_8245925, EPI_ISL_8245928			
see above	Robert Koch-Institut ZBS1 (Zentrum für biologische Gefahren und spezielle Pathogene hochpathogene Viren)	Robert Koch Institute	
EPI_ISL_7748515, EPI_ISL_7749103, EPI_ISL_7749227, EPI_ISL_7749359, EPI_ISL_7754464, EPI_ISL_7754468, EPI_ISL_7754475, EPI_ISL_7754513, EPI_ISL_7754608, EPI_ISL_7754612, EPI_ISL_7754809, EPI_ISL_7754894, EPI_ISL_7754897, EPI_ISL_7754929, EPI_ISL_7761581, EPI_ISL_7761642, EPI_ISL_7762074, EPI_ISL_7762319, EPI_ISL_7762857, EPI_ISL_8217835, EPI_ISL_8217979, EPI_ISL_8218057, EPI_ISL_8218065, EPI_ISL_8218193, EPI_ISL_8225111, EPI_ISL_8225123, EPI_ISL_8225155, EPI_ISL_8225228, EPI_ISL_8225292, EPI_ISL_8225303, EPI_ISL_8225382, EPI_ISL_8225495, EPI_ISL_8225506, EPI_ISL_8225576, EPI_ISL_8225632, EPI_ISL_8225661, EPI_ISL_8226682, EPI_ISL_8226705, EPI_ISL_8226725, EPI_ISL_8226731, EPI_ISL_8226758, EPI_ISL_8226789, EPI_ISL_8226913, EPI_ISL_8227179, EPI_ISL_8227180, EPI_ISL_8227204, EPI_ISL_8230518, EPI_ISL_8230581, EPI_ISL_8230909, EPI_ISL_8230945, EPI_ISL_8230951, EPI_ISL_8230990, EPI_ISL_8231056, EPI_ISL_8231078, EPI_ISL_8231264, EPI_ISL_8232144, EPI_ISL_8232670, EPI_ISL_8233059, EPI_ISL_8233298, EPI_ISL_8233503, EPI_ISL_8233577, EPI_ISL_8233584, EPI_ISL_8243242, EPI_ISL_8243346, EPI_ISL_8244142, EPI_ISL_8244144, EPI_ISL_8244167, EPI_ISL_8244281, EPI_ISL_8244289, EPI_ISL_8244309, EPI_ISL_8244484, EPI_ISL_8244583, EPI_ISL_8244626, EPI_ISL_8244810, EPI_ISL_8247618, EPI_ISL_8247642, EPI_ISL_8247798, EPI_ISL_8247814, EPI_ISL_8247846, EPI_ISL_8247891, EPI_ISL_8247959, EPI_ISL_8248047, EPI_ISL_8410811, EPI_ISL_8411070, EPI_ISL_8411092, EPI_ISL_8411097, EPI_ISL_8411100, EPI_ISL_8411114, EPI_ISL_8411125, EPI_ISL_8411132, EPI_ISL_8411148, EPI_ISL_8411172, EPI_ISL_8411188, EPI_ISL_8411201, EPI_ISL_8411242, EPI_ISL_8411249, EPI_ISL_8411280, EPI_ISL_8411291, EPI_ISL_8411302, EPI_ISL_8411310, EPI_ISL_8411330, EPI_ISL_8411332, EPI_ISL_8411340, EPI_ISL_8411362, EPI_ISL_8411363, EPI_ISL_8411390, EPI_ISL_8411402, EPI_ISL_8411407, EPI_ISL_8411431, EPI_ISL_8411452, EPI_ISL_8411454, EPI_ISL_8411456, EPI_ISL_8411472, EPI_ISL_8411474, EPI_ISL_8411485, EPI_ISL_8411495, EPI_ISL_8411499, EPI_ISL_8411517, EPI_ISL_8411518, EPI_ISL_8411519, EPI_ISL_8411520, EPI_ISL_8411600, EPI_ISL_8411621, EPI_ISL_8411632, EPI_ISL_8411711, EPI_ISL_8411716, EPI_ISL_8663413, EPI_ISL_8667437, EPI_ISL_8667490, EPI_ISL_8667534, EPI_ISL_8667541, EPI_ISL_8667558, EPI_ISL_8667574, EPI_ISL_8667599, EPI_ISL_8667689, EPI_ISL_8667700, EPI_ISL_8667814, EPI_ISL_8667899, EPI_ISL_8667922, EPI_ISL_8667953, EPI_ISL_8667956, EPI_ISL_8667978, EPI_ISL_8668005, EPI_ISL_8674537, EPI_ISL_8674626, EPI_ISL_8674750, EPI_ISL_8674758, EPI_ISL_8674777, EPI_ISL_8674812, EPI_ISL_8674821, EPI_ISL_8674828, EPI_ISL_8674878, EPI_ISL_8674881, EPI_ISL_8676318, EPI_ISL_8676319, EPI_ISL_8676417, EPI_ISL_8676450, EPI_ISL_8676451, EPI_ISL_8676472, EPI_ISL_8683727			
see above	SYNLAB MVZ Berlin	Robert Koch Institute	

Supplementary table S3. Parameter estimates for the analysis presented in the main text and the three sensitivity analyses. Median and 95% highest posterior density interval (HPDI) of parameter posterior distributions: Sensitivity analysis 1 (S1) fixes the become non-infectious rate to 40.5 days^{-1} for Omicron clusters. Sensitivity analysis 2 (S2) fixes the sampling proportion to 4% for all clusters. Sensitivity analysis 3 (S3) excludes all sequences that are not marked as being part of the 'random sampling' effort. The last two columns we find the 95% HPDIs of the inferred transmission rate ratio to overlap between all sensitivity analyses.

	S1: median	S1: 95% HPDI	S2: median	S2: 95% HPDI	S3: median	S3: 95% HPDI	median	95% HPDI
posterior	−5.2752e+06	[−5.2752e+06, −5.2751e+06]	−5.2753e+06	[−5.2753e+06, −5.2752e+06]	−3.9862e+06	[−3.9863e+06, −3.9862e+06]	−5.2752e+06	[−5.2752e+06, −5.2751e+06]
likelihood	−5.2803e+06	[−5.2804e+06, −5.2803e+06]	−5.2804e+06	[−5.2804e+06, −5.2804e+06]	−3.9896e+06	[−3.9896e+06, −3.9895e+06]	−5.2803e+06	[−5.2804e+06, −5.2803e+06]
prior	5.1619e+03	[5.1157e+03, 5.2094e+03]	5.1193e+03	[5.0651e+03, 5.1771e+03]	3.3629e+03	[3.3287e+03, 3.4004e+03]	5.1548e+03	[5.1073e+03, 5.1996e+03]
transition/transversion bias κ	4.7724e+00	[4.3048e+00, 5.2732e+00]	4.7691e+00	[4.2261e+00, 5.2262e+00]	4.5840e+00	[4.0466e+00, 5.1432e+00]	4.7721e+00	[4.3390e+00, 5.3031e+00]
become non−infectious rate δ_{Δ}	3.6500e+01	–	3.6500e+01	–	3.6500e+01	–	3.6500e+01	–
become non−infectious rate δ_{O}	4.0500e+01	–	NA	NA	NA	NA	NA	NA
reproductive number $R_{e,1}$	1.2747e+00	[1.2040e+00, 1.3480e+00]	1.5945e+00	[1.5202e+00, 1.6775e+00]	1.3226e+00	[1.2412e+00, 1.4072e+00]	1.2754e+00	[1.2034e+00, 1.3487e+00]
reproductive number $R_{e,2}$	1.0461e+00	[9.4638e−01, 1.1603e+00]	1.1357e+00	[1.0302e+00, 1.2453e+00]	9.5754e−01	[8.2441e−01, 1.0823e+00]	1.0496e+00	[9.4508e−01, 1.1590e+00]
lambda ratio $r_{\lambda,\Delta}$	1.0000e+00	–	1.0000e+00	–	1.0000e+00	–	1.0000e+00	–
lambda ratio $r_{\lambda,O}$	1.8299e+00	[1.6325e+00, 2.0576e+00]	1.8552e+00	[1.6588e+00, 2.0663e+00]	1.9711e+00	[1.6838e+00, 2.3031e+00]	1.9335e+00	[1.7243e+00, 2.1749e+00]
sampling proportion $s_{\Delta,1}$	0.0000e+00	–	0.0000e+00	–	0.0000e+00	–	0.0000e+00	–
sampling proportion $s_{\Delta,2}$	1.1780e−01	[1.0156e−01, 1.3492e−01]	4.0000e−02	–	1.0719e−01	[9.0789e−02, 1.2340e−01]	1.1771e−01	[1.0170e−01, 1.3512e−01]
sampling proportion $s_{O,1}$	0.0000e+00	–	0.0000e+00	–	0.0000e+00	–	0.0000e+00	–
sampling proportion $s_{O,2}$	5.5331e−02	[4.3859e−02, 6.7997e−02]	4.0000e−02	–	5.1991e−02	[3.9791e−02, 6.4791e−02]	5.7886e−02	[4.6350e−02, 7.0272e−02]
frequency parameter π_A	2.9874e−01	[2.9829e−01, 2.9915e−01]	2.9875e−01	[2.9829e−01, 2.9921e−01]	2.9874e−01	[2.9823e−01, 2.9927e−01]	2.9876e−01	[2.9831e−01, 2.9915e−01]
frequency parameter π_C	1.8337e−01	[1.8296e−01, 1.8375e−01]	1.8336e−01	[1.8302e−01, 1.8383e−01]	1.8333e−01	[1.8291e−01, 1.8376e−01]	1.8335e−01	[1.8296e−01, 1.8372e−01]
frequency parameter π_G	1.9597e−01	[1.9553e−01, 1.9633e−01]	1.9594e−01	[1.9558e−01, 1.9636e−01]	1.9593e−01	[1.9545e−01, 1.9635e−01]	1.9597e−01	[1.9550e−01, 1.9628e−01]
frequency parameter π_T	3.2194e−01	[3.2151e−01, 3.2237e−01]	3.2193e−01	[3.2149e−01, 3.2239e−01]	3.2199e−01	[3.2149e−01, 3.2251e−01]	3.2192e−01	[3.2146e−01, 3.2237e−01]
gamma shape parameter	7.1591e−01	[1.2930e−03, 2.8524e+00]	6.8034e−01	[1.3619e−03, 3.0992e+00]	6.9619e−01	[1.1105e−03, 2.9826e+00]	7.1589e−01	[1.0626e−03, 2.8505e+00]
clock rate	8.0000e−04	–	8.0000e−04	–	8.0000e−04	–	8.0000e−04	–

Supplementary table S4. Parameter estimates for the analysis presented in the main text and two other cluster size inclusion thresholds. Median and 95% highest posterior density interval (HPDI) of parameter posterior distributions for analysis with three different minimum cluster size thresholds: 4, 10 and 20. The first corresponds to the analysis presented in the main text. We find the 95% HPDIs of the inferred transmission rate ratio to overlap between all sensitivity analyses.

	min10: median	min10: 95% HPDI	min20: median	min20: 95% HPDI	min4: median	min4: 95% HPDI
posterior	−1.0511e+06	[−1.0511e+06, −1.0510e+06]	−4.0525e+05	[−4.0530e+05, −4.0521e+05]	−5.2752e+06	[−5.2752e+06, −5.2751e+06]
likelihood	−1.0540e+06	[−1.0541e+06, −1.0540e+06]	−4.0730e+05	[−4.0733e+05, −4.0728e+05]	−5.2803e+06	[−5.2804e+06, −5.2803e+06]
prior	2.9706e+03	[2.9193e+03, 3.0212e+03]	2.0477e+03	[2.0049e+03, 2.0942e+03]	5.1550e+03	[5.1090e+03, 5.1997e+03]
transition/transversion bias κ	4.9806e+00	[4.2820e+00, 5.7217e+00]	5.2499e+00	[4.2329e+00, 6.3939e+00]	4.7581e+00	[4.2823e+00, 5.2836e+00]
become non−infectious rate δ	3.6500e+01	–	3.6500e+01	–	3.6500e+01	–
reproductive number $R_{e,1}$	1.6986e+00	[1.5489e+00, 1.8623e+00]	1.7005e+00	[1.4712e+00, 1.9648e+00]	1.2760e+00	[1.2054e+00, 1.3482e+00]
reproductive number $R_{e,2}$	9.4478e−01	[8.0075e−01, 1.1097e+00]	1.2637e+00	[1.0284e+00, 1.5059e+00]	1.0463e+00	[9.4270e−01, 1.1547e+00]
lambda ratio $r_{\lambda,\Delta}$	1.0000e+00	–	1.0000e+00	–	1.0000e+00	–
lambda ratio $r_{\lambda,O}$	2.1222e+00	[1.7563e+00, 2.5176e+00]	1.5949e+00	[1.3117e+00, 1.9372e+00]	1.9430e+00	[1.7273e+00, 2.1844e+00]
sampling proportion $s_{\Delta,1}$	0.0000e+00	–	0.0000e+00	–	0.0000e+00	–
sampling proportion $s_{\Delta,2}$	6.8951e−02	[5.4611e−02, 8.3714e−02]	4.9400e−02	[3.6751e−02, 6.2933e−02]	1.1763e−01	[1.0043e−01, 1.3422e−01]
sampling proportion $s_{O,1}$	0.0000e+00	–	0.0000e+00	–	0.0000e+00	–
sampling proportion $s_{O,2}$	5.9574e−02	[4.7200e−02, 7.3183e−02]	5.9152e−02	[4.6461e−02, 7.1831e−02]	5.7782e−02	[4.6230e−02, 7.0709e−02]
frequency parameter π_A	2.9870e−01	[2.9767e−01, 2.9972e−01]	2.9883e−01	[2.9720e−01, 3.0043e−01]	2.9875e−01	[2.9833e−01, 2.9927e−01]
frequency parameter π_C	1.8341e−01	[1.8260e−01, 1.8438e−01]	1.8335e−01	[1.8200e−01, 1.8477e−01]	1.8335e−01	[1.8300e−01, 1.8377e−01]
frequency parameter π_G	1.9593e−01	[1.9505e−01, 1.9679e−01]	1.9590e−01	[1.9447e−01, 1.9733e−01]	1.9595e−01	[1.9558e−01, 1.9634e−01]
frequency parameter π_T	3.2198e−01	[3.2098e−01, 3.2308e−01]	3.2192e−01	[3.2020e−01, 3.2357e−01]	3.2194e−01	[3.2149e−01, 3.2239e−01]
gamma shape parameter	7.1461e−01	[1.5574e−03, 2.9966e+00]	6.8751e−01	[1.0307e−03, 3.0540e+00]	6.9335e−01	[1.9212e−03, 3.2363e+00]
clock rate	8.0000e−04	–	8.0000e−04	–	8.0000e−04	–

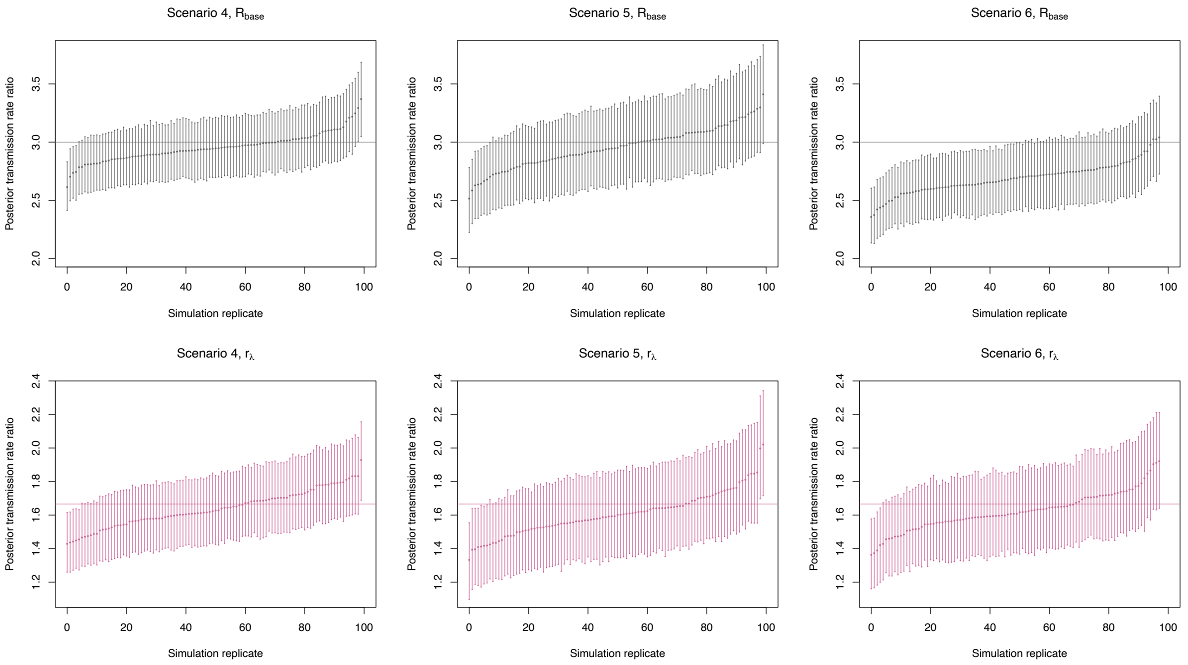

Supplementary figure S1. Estimated posterior distributions for R_{base} (upper panel) and $r_{\lambda,high}$ (lower panel) for simulation scenarios 4–6 (from left to right). For each scenario 100 parallel simulations were performed and only those replicates for which all parameters yielded ESS values >200 are included. Bars demonstrate 95% highest posterior density intervals for individual simulation replicates, points indicating median estimates. Horizontal lines represent true values (i.e. $R_{base} = 3.0$ and $r_{\lambda,high} = 1.666$).

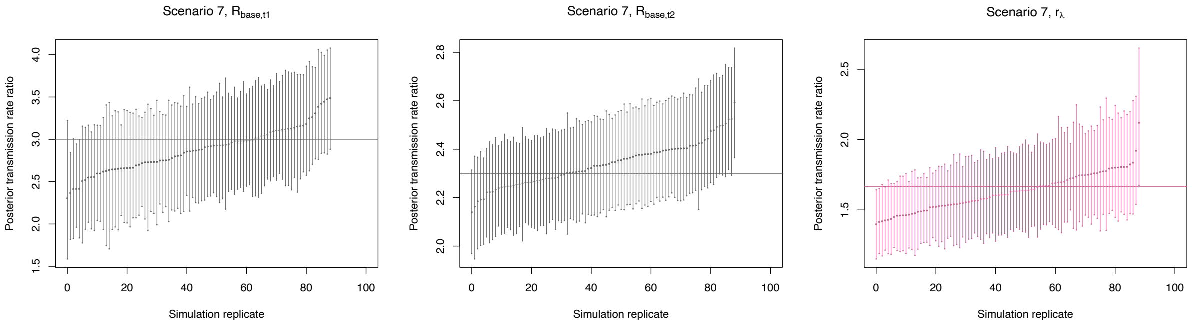

Supplementary figure S2. Estimated posterior distributions for $R_{base,t1}$, $R_{base,t2}$ and $r_{\lambda,high}$ for simulation scenario 7. 100 parallel simulations were performed and only those replicates for which all parameters yielded ESS values >200 are included. Bars demonstrate 95% highest posterior density intervals for individual simulation replicates, points indicating median estimates. Horizontal lines represent true values (i.e. $R_{base,t1} = 3.0$, $R_{base,t2} = 2.3$ and $r_{\lambda,high} = 1.666$).

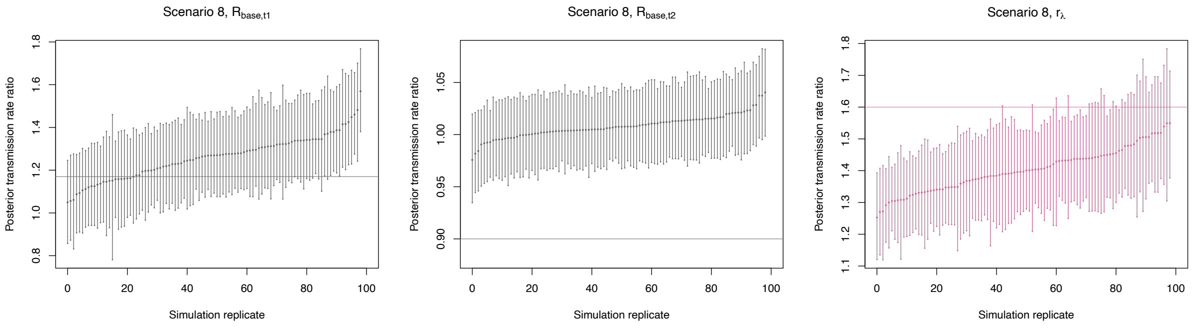

Supplementary figure S3. Estimated posterior distributions for $R_{base,t1}$, $R_{base,t2}$ and $r_{\lambda,high}$ for simulation scenario 8. 100 parallel simulations were performed and only those replicates for which all parameters yielded ESS values >200 are included. Bars demonstrate 95% highest posterior density intervals for individual simulation replicates, points indicating median estimates. Horizontal lines represent true values (i.e. $R_{base,t1} = 1.17$, $R_{base,t2} = 0.9$ and $r_{\lambda,high} = 1.6$).

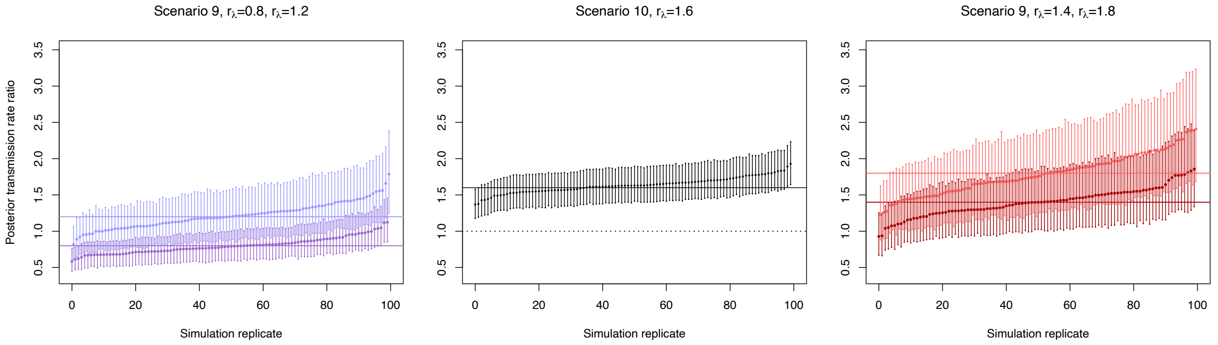

Supplementary figure S4. Comparison of estimated posterior distributions for $r_{\lambda,low}$ and $r_{\lambda,high}$ between simulation scenarios 9 and 10. In the simulation scenario 9 transmission rate ratio was inferred independently for all transmission trees whereas in simulation scenario 10 transmission rate ratio was inferred jointly for trees simulated with $r_{\lambda,low}$ (i.e. $r_{\lambda,low} = 0.8, 0.9, 1.0, 1.1, 1.2$) and jointly for trees simulated with $r_{\lambda,high}$ (i.e. $r_{\lambda,high} = 1.4, 1.5, 1.6, 1.7, 1.8$). Left and right panels represent results from simulation scenario 9. On the left panel posterior distributions from scenario 9 are presented for the trees with lowest and highest transmission rate ratio of the $r_{\lambda,low}$ class (with lilac colour $r_{\lambda,low} = 0.8$ and with light blue colour $r_{\lambda,low} = 1.2$). Similarly, on the right panel posterior distributions from scenario 9 are presented for the trees with lowest and highest transmission rate ratio of the $r_{\lambda,high}$ class (with red colour $r_{\lambda,high} = 1.4$ and with orange colour $r_{\lambda,high} = 1.8$). The middle panel demonstrates the posterior distributions for $r_{\lambda,high}$ obtained from simulation scenario 10. In each panel, horizontal solid lines represent the true values. In the middle panel, dashed horizontal line indicates the true value for $r_{\lambda,low}$ class. As for the Bayesian inference $r_{\lambda,low}$ parameter was fixed to its true value of 1.0, no posterior estimates were obtained.

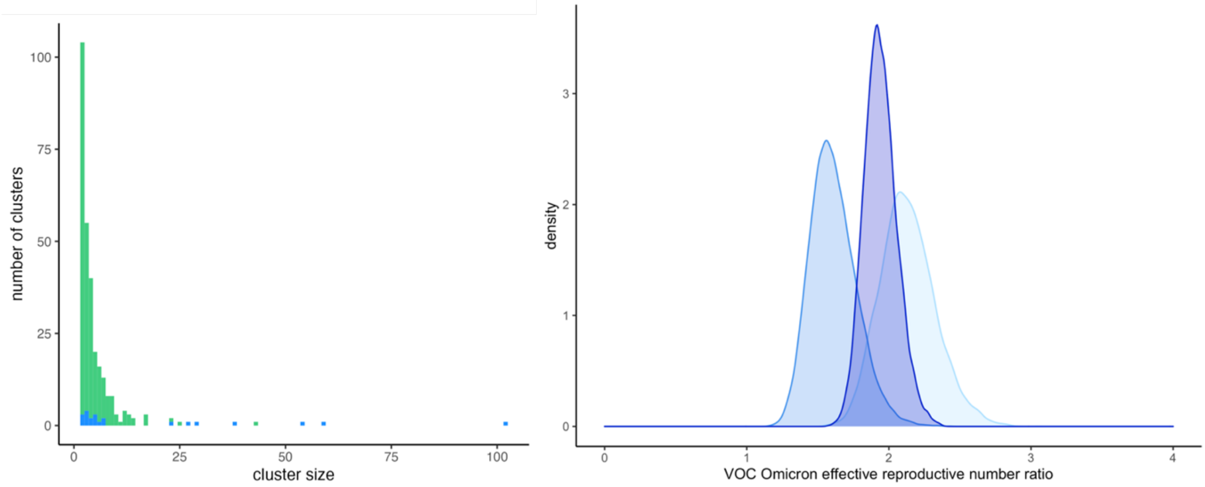

Supplementary figure S5. Impact of cluster size distribution on SARS-CoV-2 data analysis. Left: Histogram of cluster sizes, i.e. number of included sequences. Delta-associated clusters are shown in green, Omicron-associated clusters in blue. Right: Sensitivity of the inferred transmission rate ratio with regard to the size of the smallest included clusters. Light blue shows the result from clusters of at least 10, blue of at least 20 and dark blue of 4, *cf.* Supplementary Table 4.

References

Attwood SW, Hill SC, Aanensen DM, Connor TR, Pybus OG. 2022. Phylogenetic and phylodynamic approaches to understanding and combating the early SARS-CoV-2 pandemic. *Nature Reviews Genetics* 23:547–562.

Byrne AW, McEvoy D, Collins AB, Hunt K, Casey M, Barber A, Butler F, Griffin J, Lane EA, McAloon C, et al. 2020. Inferred duration of infectious period of SARS-CoV-2: rapid scoping review and analysis of available evidence for asymptomatic and symptomatic COVID-19 cases. *BMJ Open* 10:e039856.

Geoghegan JL, Ren X, Storey M, Hadfield J, Jelley L, Jefferies S, Sherwood J, Paine S, Huang S, Douglas J, et al. 2020. Genomic epidemiology reveals transmission patterns and dynamics of SARS-CoV-2 in Aotearoa New Zealand. *Nature Communications* 11:6351.

Ghafari M, Du Plessis L, Pybus OG, Katzourakis A. 2020. Time dependence of SARS-CoV-2 substitution rates. *Virological* [Internet]. Available from: https://virological.org/t/time-dependence-of-sars-cov-2-substitution-rates/542

Lam A, Duchene S. 2021. The Impacts of Low Diversity Sequence Data on Phylodynamic Inference during an Emerging Epidemic. *Viruses* 13:79.

Liu Y, Gayle AA, Wilder-Smith A, Rocklöv J. 2020. The reproductive number of COVID-19 is higher compared to SARS coronavirus. *Journal of Travel Medicine* 27:1–4.

Liu Y, Rocklöv J. 2021. The reproductive number of the Delta variant of SARS-CoV-2 is far higher compared to the ancestral SARS-CoV-2 virus. *Journal of Travel Medicine*:1–3.

Markov PV, Ghafari M, Beer M, Lythgoe K, Simmonds P, Stilianakis NI, Katzourakis A. 2023. The evolution of SARS-CoV-2. *Nature Reviews Microbiology* 21:361–379.

Nadeau SA, Vaughan TG, Scire J, Huisman JS, Stadler T. 2021. The origin and early spread of SARS-CoV-2 in Europe. *Proceedings of the National Academy of Sciences of the United States of America* 118:1–8.

NIID. 2022. Active epidemiological investigation on SARS-CoV-2 infection caused by Omicron variant (Pango lineage B.1.1.529) in Japan: preliminary report on infectious period. National Institute of Infectious Diseases Disease Control and Prevention Center, National Center for Global Health and Medicine. Available from: https://www.niid.go.jp/niid/en/2019-ncov-e/10884-covid19-66-en.html

du Plessis L, McCrone JT, Zarebski AE, Hill V, Ruis C, Gutierrez B, Raghwani J, Ashworth J, Colquhoun R, Connor TR, et al. 2021. Establishment and lineage dynamics of the SARS-CoV-2 epidemic in the UK. *Science* 371:708–712.

Ragonnet-Cronin M, Hodcroft E, Hué S, Fearnhill E, Delpech V, Brown AJL, Lycett S. 2013. Automated analysis of phylogenetic clusters. *BMC Bioinformatics* 14:1–10.

Seemann T, Lane CR, Sherry NL, Duchene S, Gonçalves da Silva A, Caly L, Sait M, Ballard SA, Horan K, Schultz MB, et al. 2020. Tracking the COVID-19 pandemic in Australia using genomics. *Nature Communications* 11:1–9.

Siedner MJ, Boucau J, Gilbert RF, Uddin R, Luu J, Haneuse S, Vyas T, Reynolds Z, Iyer S, Chamberlin GC, et al. 2022. Duration of viral shedding and culture positivity with postvaccination SARS-CoV-2 delta variant infections. *JCI Insight* 7.

Stadler T, Kühnert D, Bonhoeffer S, Drummond AJ. 2013. Birth-death skyline plot reveals temporal changes of epidemic spread in HIV and hepatitis C virus (HCV). *Proceedings of the National Academy of Sciences of the United States of America* 110:228–233.

Tay JH, Porter AF, Wirth W, Duchene S. 2022. The Emergence of SARS-CoV-2 Variants of Concern Is Driven by Acceleration of the Substitution Rate. *Molecular Biology and Evolution* 39:1–9.
